# CFD study on NACA 4415 airfoil implementing spherical and sinusoidal Tubercle Leading Edge

**DOI:** 10.1371/journal.pone.0183456

**Published:** 2017-08-29

**Authors:** S. M. A. Aftab, K. A. Ahmad

**Affiliations:** 1 Dept of Aerospace Engineering, Universiti Putra Malaysia, Selangor, 43400, Malaysia; 2 Mechanical Engineering Department, College of Engineering, King Saud University, P.O. Box 800, Riyadh 11421, Saudi Arabia; Coastal Carolina University, UNITED STATES

## Abstract

The Humpback whale tubercles have been studied for more than a decade. Tubercle Leading Edge (TLE) effectively reduces the separation bubble size and helps in delaying stall. They are very effective in case of low Reynolds number flows. The current Computational Fluid Dynamics (CFD) study is on NACA 4415 airfoil, at a Reynolds number 120,000. Two TLE shapes are tested on NACA 4415 airfoil. The tubercle designs implemented on the airfoil are sinusoidal and spherical. A parametric study is also carried out considering three amplitudes (0.025c, 0.05c and 0.075c), the wavelength (0.25c) is fixed. Structured mesh is utilized to generate grid and Transition SST turbulence model is used to capture the flow physics. Results clearly show spherical tubercles outperform sinusoidal tubercles. Furthermore experimental study considering spherical TLE is carried out at Reynolds number 200,000. The experimental results show that spherical TLE improve performance compared to clean airfoil.

## 1. Introduction

Biomimetics is the art of studying and applying nature inspired designs in the field of engineering. Research utilizing Humpback whale tubercles has gained popularity over the past decade. Implementing tubercle design has shown to improve airfoil performance, dratstically reduce aeroacoustic noise and seperation bubble [1–6]. A detailed review on application of Humpback whale flipper design on various airfoils has been conducted by Aftab et al., [7]. Few of the previous parametric studies conducted considering TLE are reported below Aftab et al., [7].

1. Study considering the variation of amplitude and wavelength Johari et al., [1], Zhang et al., [2, 8, 9], Lohry et al., [10] and Koun et al., [11].
2. Custodio et al., [3] studied four planform geometries: rectangular finite span, infinite span, swept and idealized flipper model.
3. Yoon et al., [12] studied the effect of waviness along the span, for 5 different waviness ratios (0.2, 0.4, 0.6, 0.8 and 1.0), in comparison with base line airfoil. Kim et al., [13] extended the previous study of Yoon et al., [12], studying the effect of 5 wavelengths ($\frac{S}{2}$,$\frac{S}{4}$,$\frac{S}{6}$,$\frac{S}{8}$ and $\frac{S}{10}$, S is span 1.5c) for fixed amplitude (0.5c).
4. Goruney and Rockwell [14], studied the effect of TLE on a swept delta wing. Chen et al., [15] investigated the effect of the tubercles on the performance of a moderately swept delta wing. Chen et al., [16] further investigated the effect of tubercles on a highly swept delta wing.
  The above mentioned researchers as well as few others, such as Custodio et al., [3], Rostamzadeh et al., [4], Miklosovic et al., [5], Borg [6], Corsini et al, [17], Zhang et al., [8, 18] and Skillen et al., [19], have worked considering only the sinusoidal tubercles designs. Only one author Gawad [20, 21] has proposed spherical TLE design. Gawad [20, 21] conducted CFD study on NACA 0012 implementing spherical TLE and reported that, it performed better compared to the sinusoidal TLE.
  Aftab et al., [22] also carried out a numerical study for low Reynolds number flow. Five turbulence models were tested in the study, out of all RANS turbulence models tested, only transition SST turbulence model is suitable to capture the transition effects. The work on transition SST turbulence model reported by Langtry and Menter [23] and Menter et al., [24] is quite accurate to capture the separation bubble and other related phenomenon for low Reynolds number flows.
  The current study is on NACA 4415 airfoil profile with sinusoidal and spherical TLE. A parametric study is carried out between the two designs by varying the tubercle amplitude, inorder to determine which design is more suitable for NACA 4415 airfoil. This study is unique as an in-depth study comparing, tubercle design has not been reported in literature. Experimental testing in wind tunnel is also carried out, based on the best tubercle design.

## 2. Geometrical design

This section deals with the creation of the geomerty using CATIA V5R21.

### 2.1 Clean airfoil

**Fig 1:**
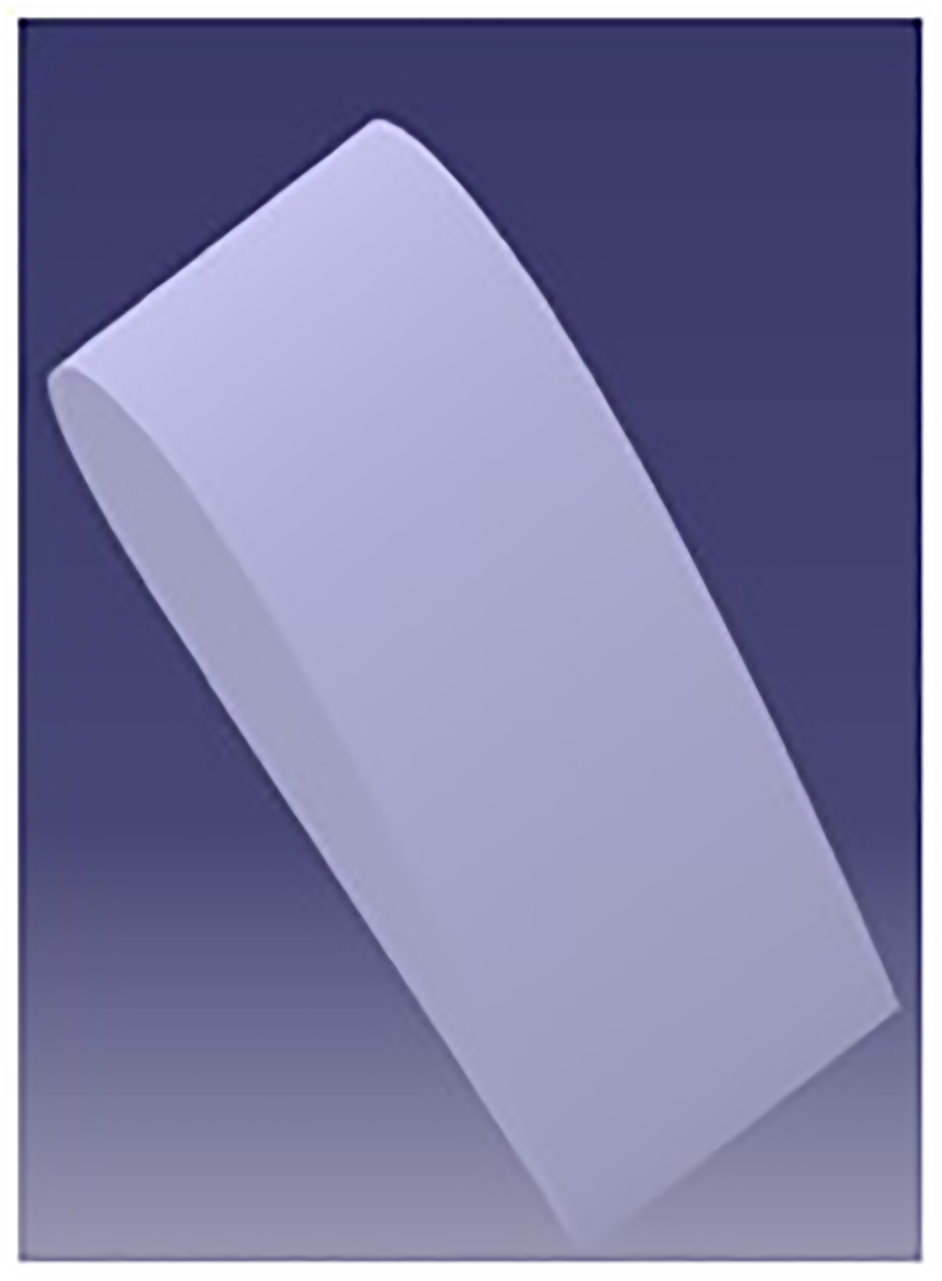
Clean leading edge NACA 4415 airfoil.

The NACA 4415 profile of unit chord (c) is created using CATIA V5R21, as shown in Fig 1.

### 2.2 Sinusoidal tubercles

**Fig 2:**
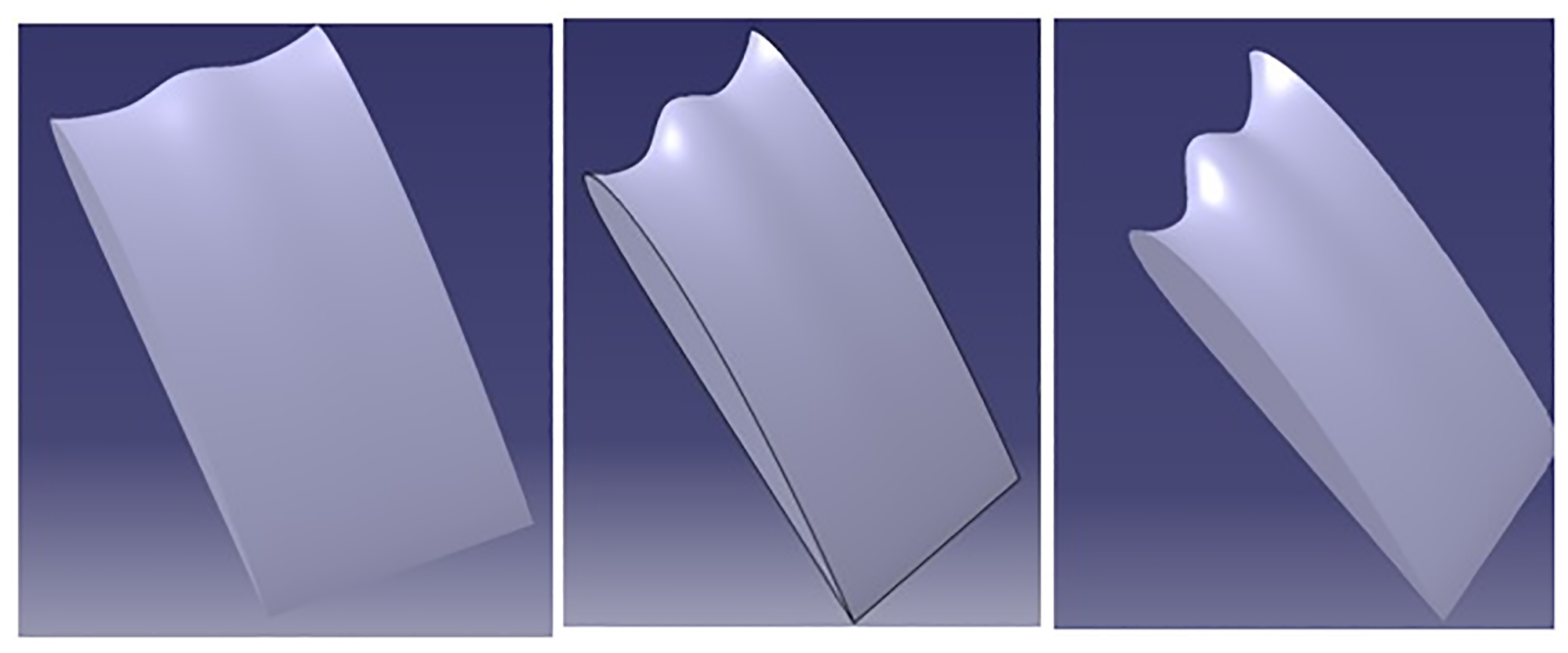
Sinusoidal tubercles with different amplitudes and constant wavelength.

The sinusoidal tubercles have been modeled using the methodology suggested by Swanson et al., [25]. Three amplitudes are modeled 0.025c, 0.05c and 0.75c and the wavelength is kept constant at 0.25c as shown in Fig 2. The airfoil coordinates are modified at the leading edge in x direction without altering the y coordinates as shown in Eq (1) and (2). The subscript old indicates the clean wing and subscripts tm refers to the location of max thickness. The Amplitude A is used to modify the leading edge by a fraction of chord length. Profiles are generated using excel and later using macros these profiles are imported into CATIA V5R21 to generate the wing surface.

$$\begin{array}{c} X_{new}=X_{old}\left(1\pm A\right)forX_{old}>X_{tm} \end{array}$$

$$\begin{array}{c} Y_{new}=Y_{old} \end{array}$$

### 2.3 Spherical tubercles

**Fig 3:**
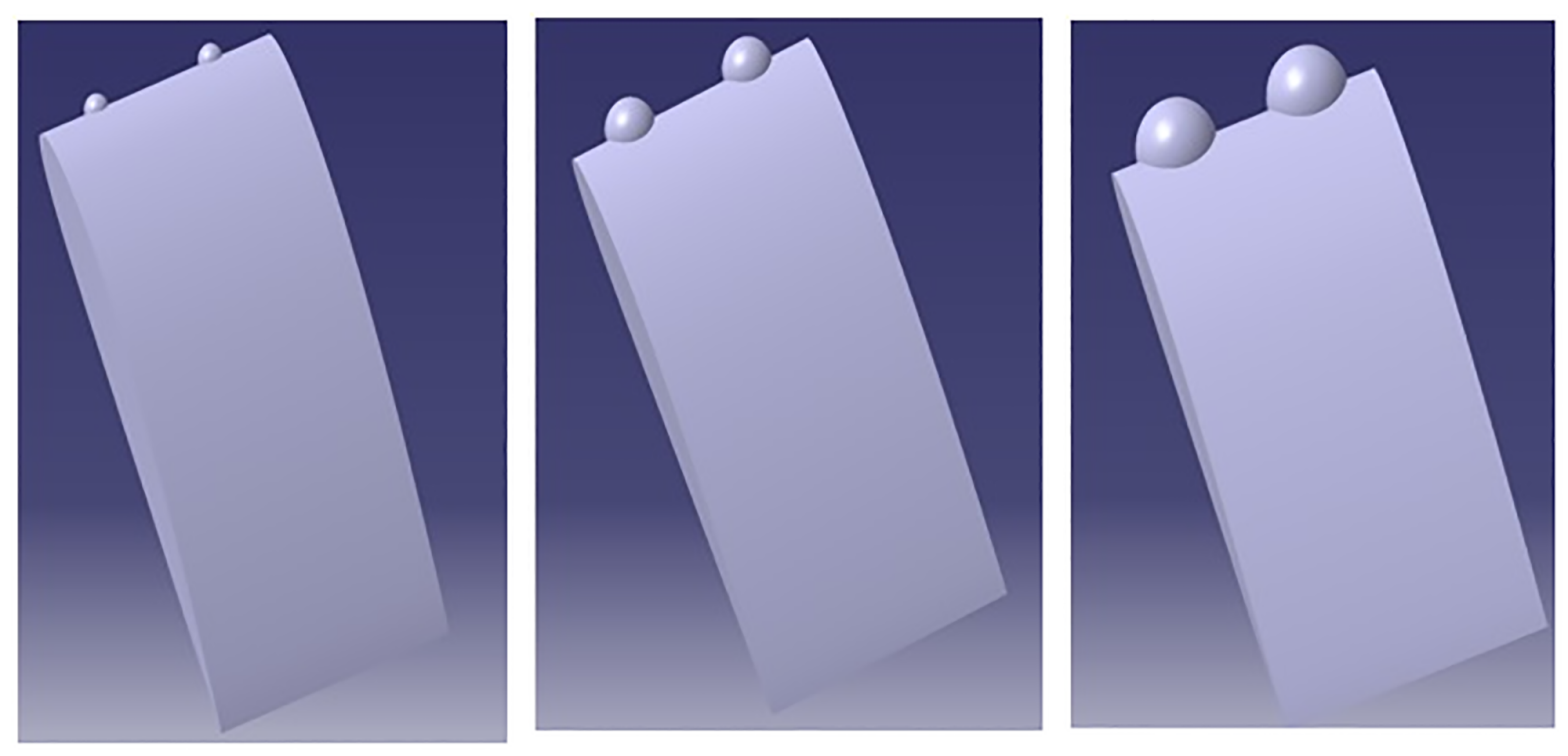
Spherical tubercles with different amplitudes and constant wavelength.

Gawad [[20, 21]] has proposed spherical design of tubercles, and also reported that the new spherical TLE design, improves the performance compared to sinusoidal TLE design. The spherical tubercles have been generated varying the radius of sphere as shown in Fig 3. Study by Aftab and Kamarul [26] noticed improvement in NACA 4415 airfoil performance implementing spherical TLE.

The major draw back noticed in the studies of Gawad [20, 21] and Aftab and Kamarul [26] was, they were based on unstructured meshing and unsuitable turbulence model. Gawad [20, 21] considered *k* − *ω* and Aftab and Kamarul [26] considered the one equation Spalart Allmaras turbulence model. The importance of selecting a proper turbulence model for low Reynolds number flows, has been discussed in detail by Aftab et al., [22].

### 2.4 Domain details

**Fig 4:**
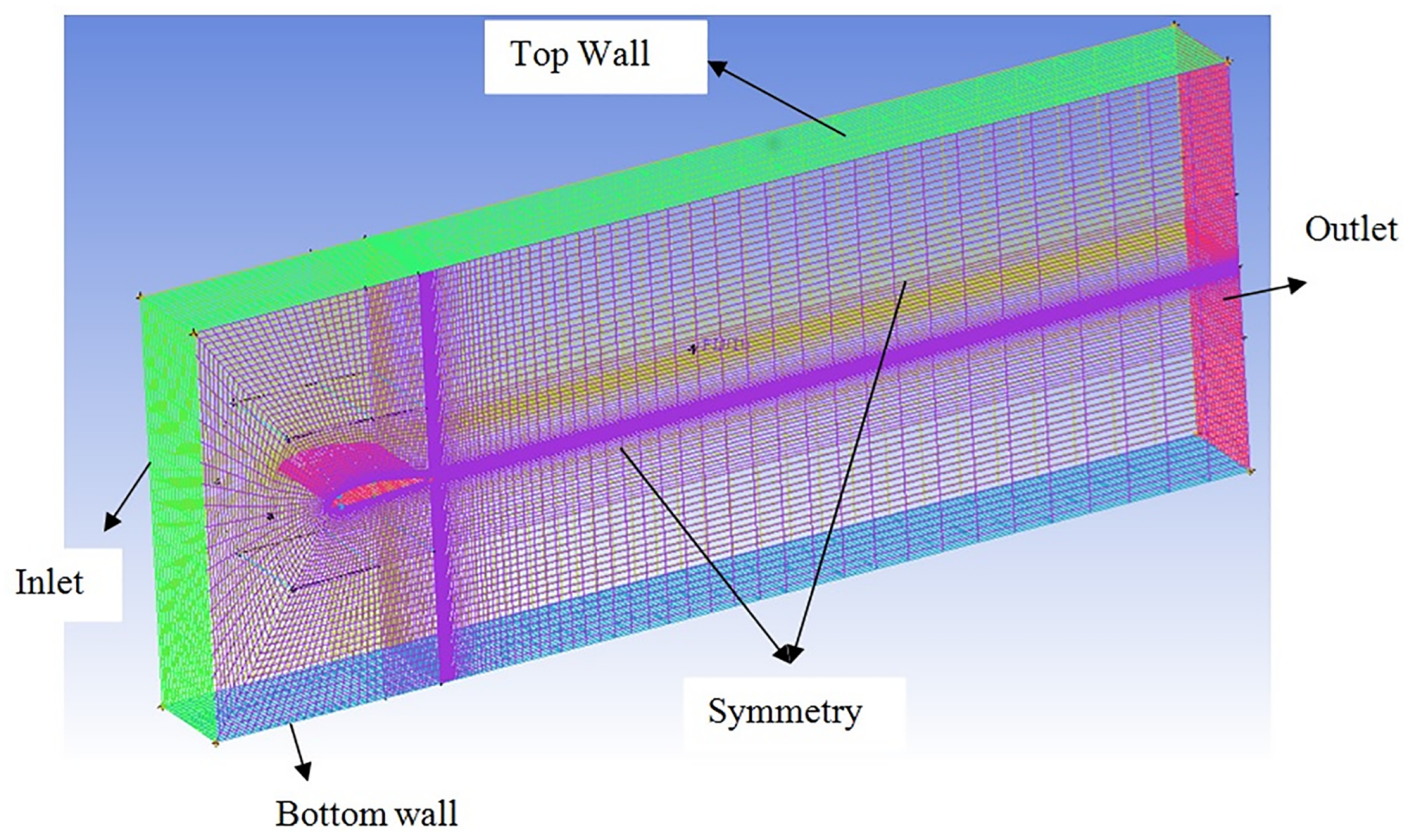
Domain with structured mesh.

**Fig 5:**
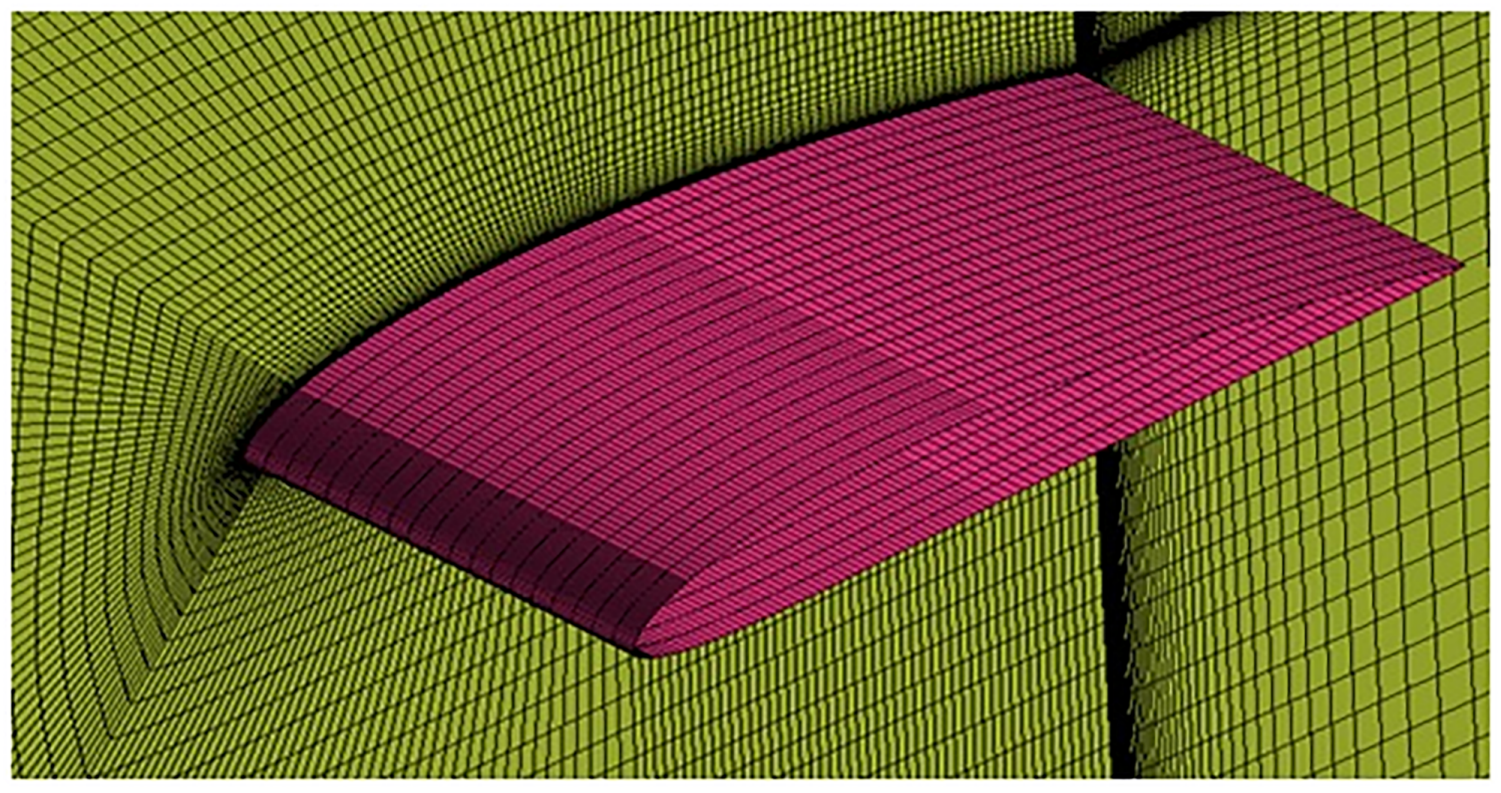
O-grid structured mesh around clean airfoil with structured mesh.

**Fig 6:**
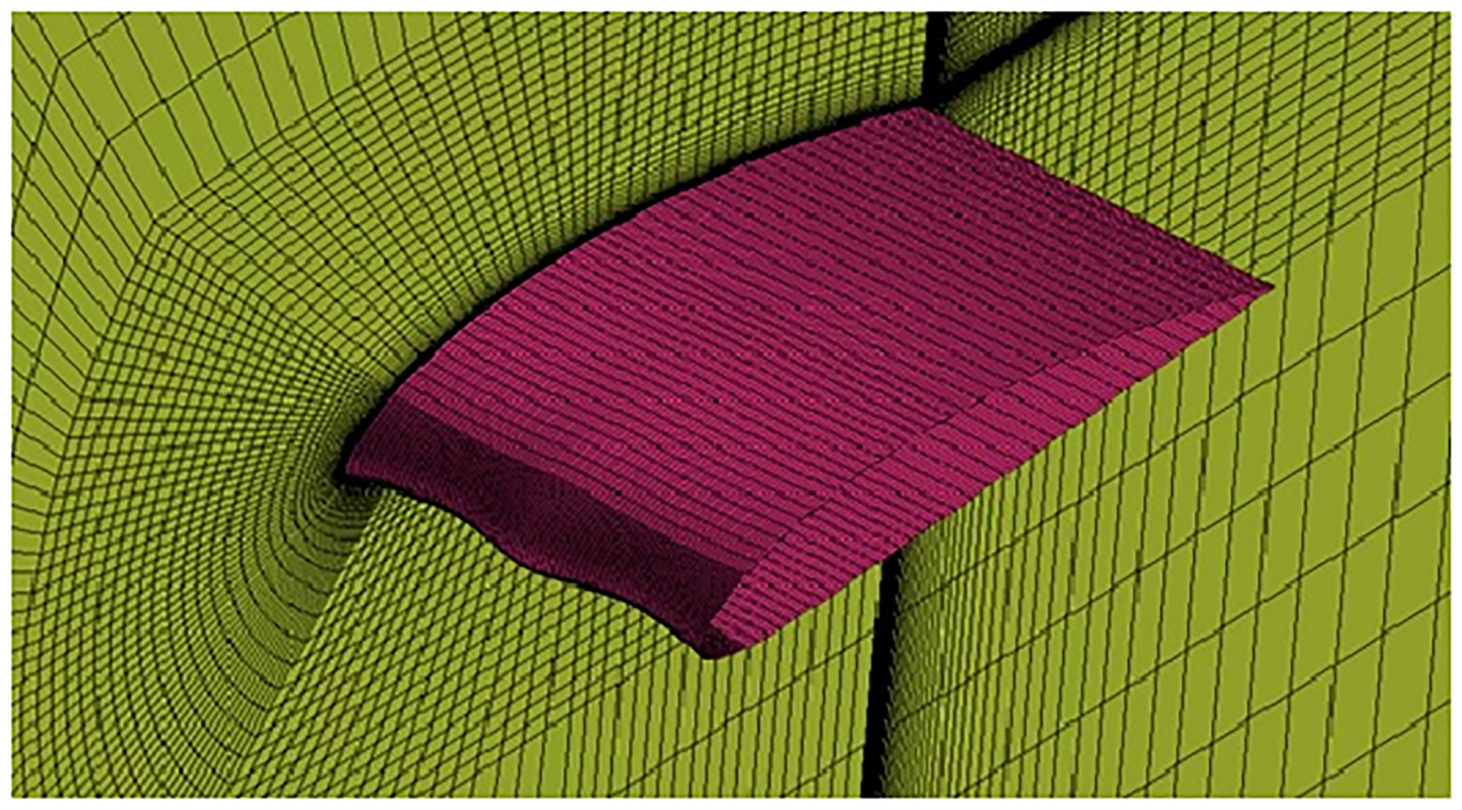
O-grid structured mesh around sinusoidal tubercle airfoil.

**Fig 7:**
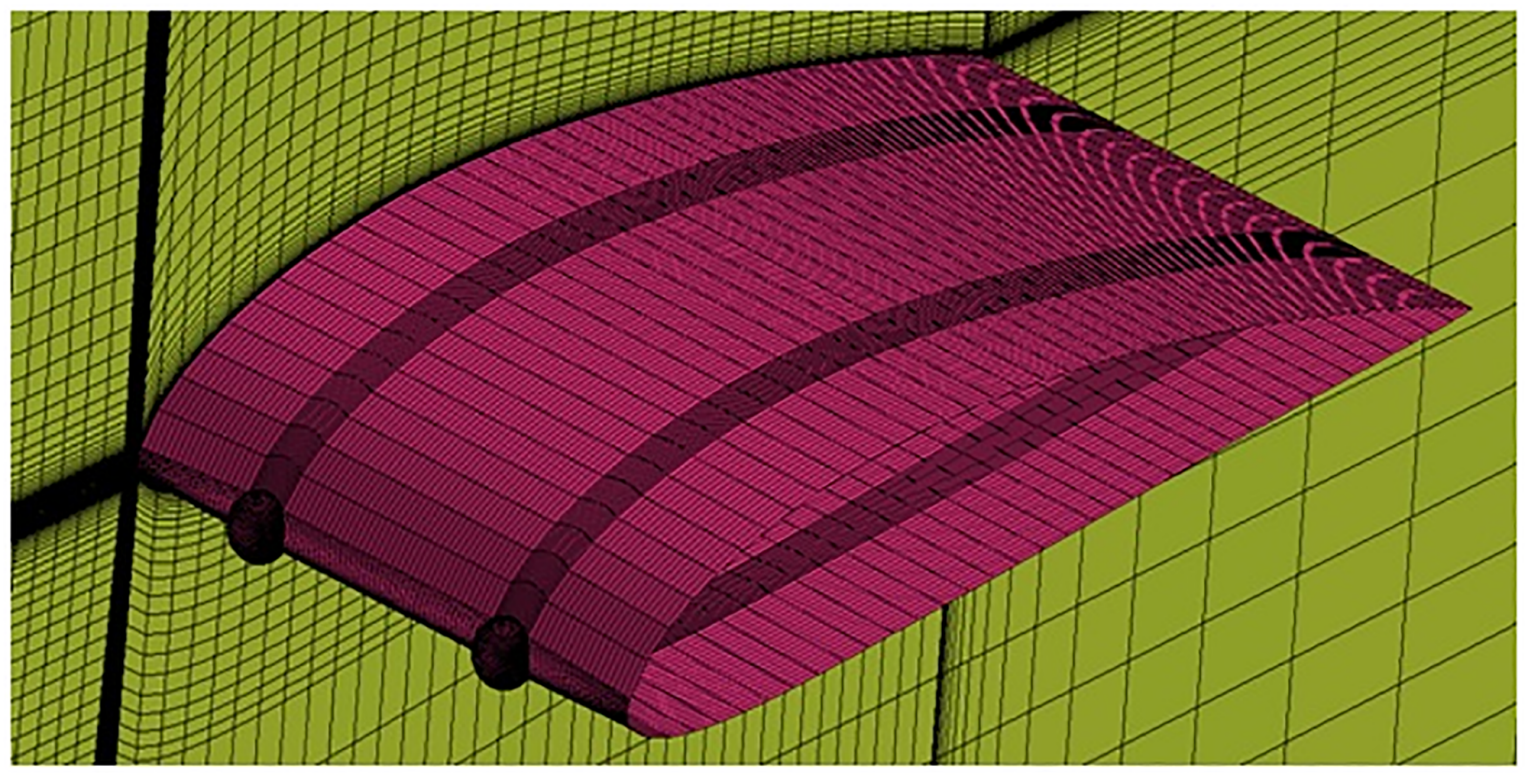
O-grid structured mesh around spherical tubercle airfoil.

A rectangular domain is created around the wing with a width equal to span of the airfoil. The inlet and outlet are kept at a distance of −1.3c and 10.3c from the airfoil leading edge. The domain is extended 2c above and below the airfoil to avoid confinement effects. The domain is similar to one used by Corsini et al., [17]. Hex mesh is generated around the airfoil. Two zones are created for meshing, the inner zone close to the airfoil is used to obtain fine grid as shown in Fig 4. The wall *y*^+^ is calculated and the estimated distance is fixed 7.9 × 10^−5^m. The domain size is maintained for clean and TLE (sinusoidal and spherical) airfoils. The mesh for clean airfoil is as shown in Fig 5. The Figs 6 and 7 show the mesh on sinusoidal and spherical tubercle airfoils. The mesh density is varied making it coarse as it goes outward away from the surface of interest.

The growth ratio around the airfoils Figs 5, 6 and 7 is maintained at 1.05 in outward direction in order to capture the Boundary Layer (BL) effects. The wall *y*^+^ <1 is also maintained which is the main requirement for capturing the transition effects.

## 3. Numerical method

This section will cover the computational aspects as well as the previous validation study.

### 3.1 Computational aspects

Commercial simulation software ANSYS was used to carry out Steady state analysis considering, transition SST turbulence model. It has been well proven and designed for low Reynolds number aerodynamic applications. Transition SST uses four transport equations to model the transition behavior which has been clearly explained in previous work of Aftab et al., [22]. The model is more accurate and the computation time required is less. SIMPLE pressure velocity coupling is implemented and the simulation is carried out for 2^*nd*^ order of accuracy and convergence criterion is set to 10^−6^.

Simulation is carried out from 0° till 18° Angle of Attack (AoA). The input parameters such as pressure, density and viscosity are considered at sea level conditions. The inlet velocity is kept at 1.76 ms^−1^ for a chord based of Reynolds number of 1.2 × 10^5^. Mesh dependence study is carried out by varying mesh sizes, results showed that 0.55 million, 1.93 million and 2.5 million, mesh size is optimum for clean, sinusoidal and spherical airfoil respectively. The methodology followed for the current mesh dependency test, is similar to the study previously reported by Aftab et al., [22]. Thus above mentioned mesh sizes are utilized for the parametric study. The results obtained are described in detail in section 4.

### 3.2 Previous validation study

**Fig 8:**
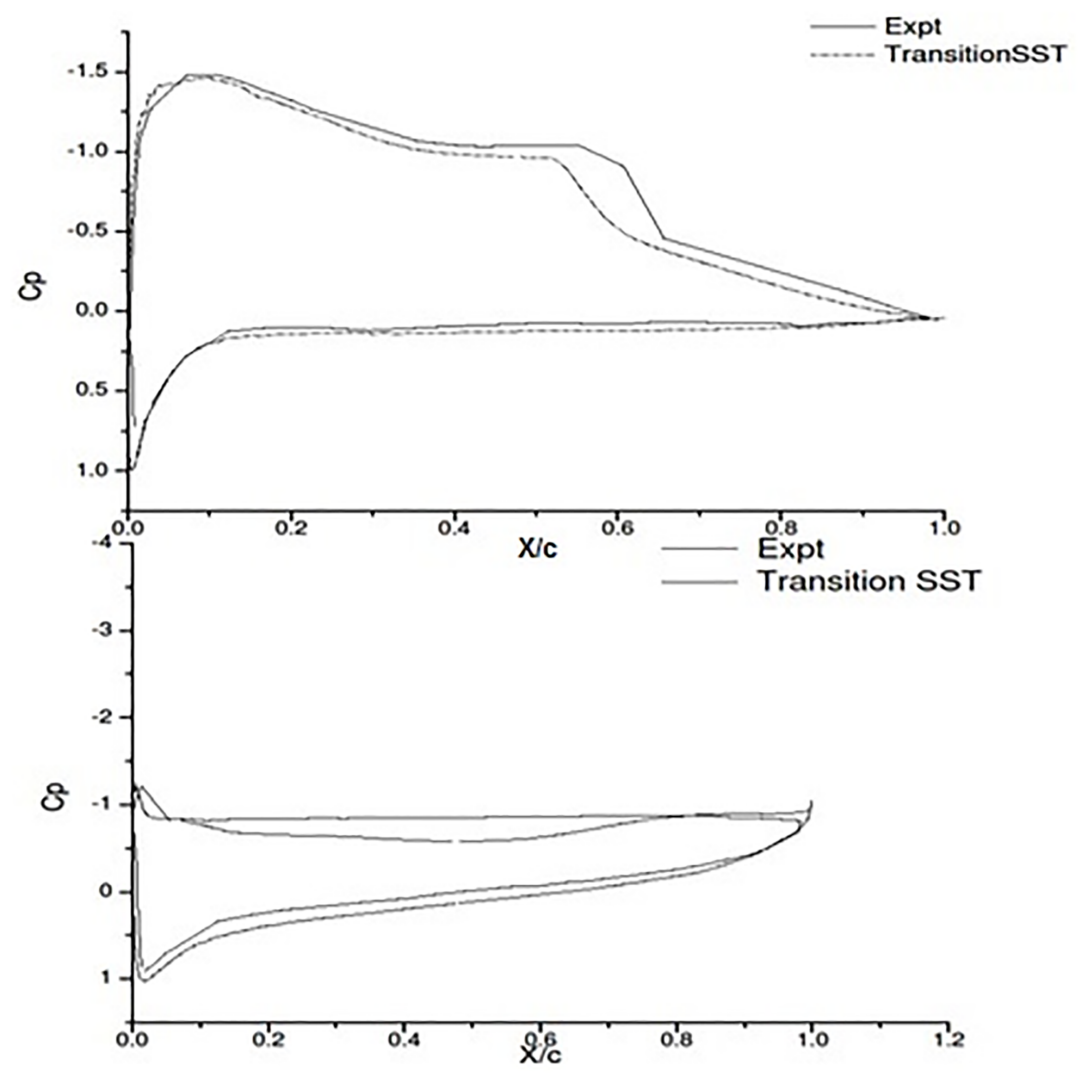
*C*_*p*_ expt and *γ*-Re_*θ*_ SST a) 6 degree AOA b) 18 degree AOA Aftab et al., [22].

Aftab et al., [22] conducted an in depth CFD validation analysis considering NACA 4415 with experimental data of Karthikayen et al., [27]. The *C*_*p*_ plot Fig 8 shows the accuracy of Transition SST Turbulence model in capturing the separation bubble at 6° and complete separation at 18°. The values of the experimental study and the CFD study of Aftab et al., [22] were found to be in good agreement.

## 4 Results and discussion

This section discusses the results of the current study. Table 1, summarizes the basic details such as TLE amplitude and Reynolds number used in the current CFD study.

**Table 1:** Parameters of the current study.

Airfoil	Amplitude	Wavelength	Reynolds number
Clean Airfoil	——-	——	1.2 × 10⌃5
Sinusoidal TLE and Spherical TLE	0.025c	0.025c	
	0.05c		
	0.075c		

### 4.1 Sinusoidal TLE

**Fig 9:**
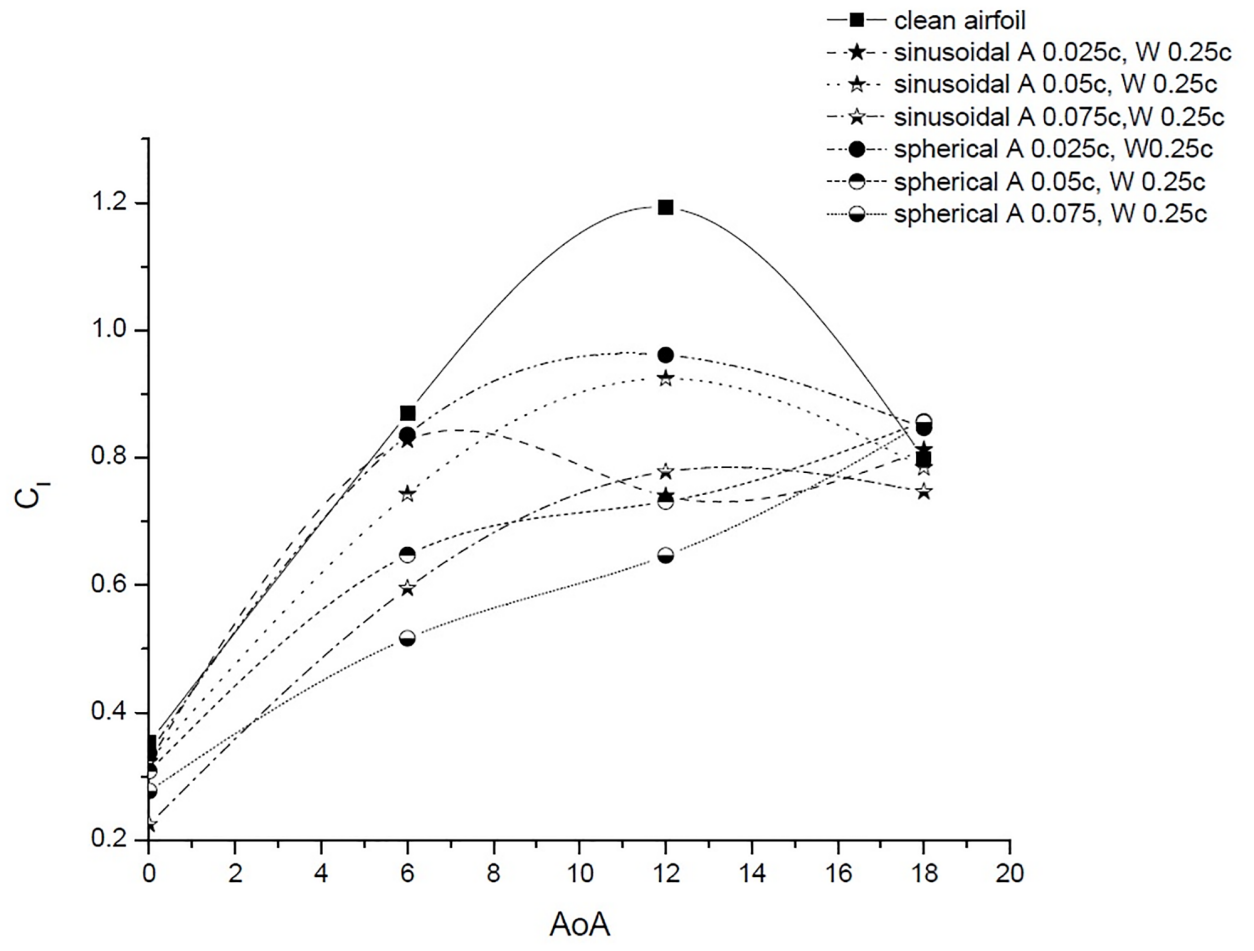
*C*_*l*_ vs. AoA.

**Fig 10:**
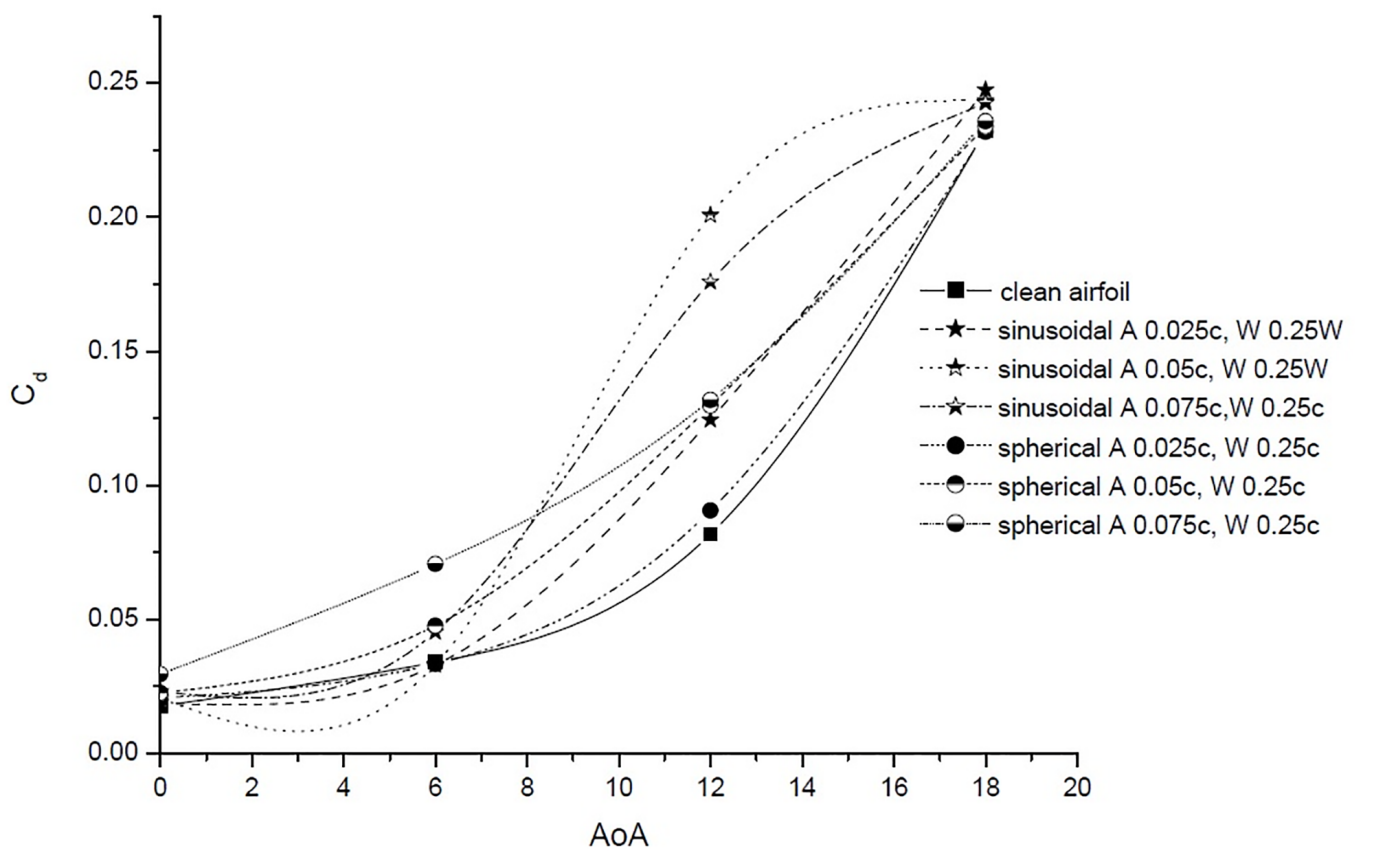
*C*_*d*_ vs. AoA.

Peformance comparision of sinusoidal TLE design with clean airfoil is discussed in this subsection. The parametric study is carried out considering sinusoidal TLE designs with three amplitudes (0.025c, 0.05c and 0.075c). The *C*_*l*_ vs AoA and *C*_*d*_ vs AoA results are as shown in Figs 9 and 10. In case of sinusoidal TLE the airfoil with tubercle amplitude 0.025c, performed better than other two sinusoidal TLE design.

Table 2 provides an in depth comparison of clean airfoil and sinusoidal TLE of amplitude 0.025c.

**Table 2:** *C*_*l*_, *C*_*d*_ and L/D for clean airfoil and sinusoidal TLE airfoil with 0.025c and 0.25c.

Airfoil	AoA	*C*_*l*_	% decrease in lift	*C*_*d*_	% increase in drag	L/D	% decrease in L/D ratio
Clean Airfoil	0	0.353		0.0177		20.00	
Sinusoidal A 0.025c and W 0.25c	0	0.323	8.58	0.0190	7.53	17.7	15.0
Clean Airfoil	6	0.870		0.0341		25.51	
Sinusoidal A 0.025c and W 0.25c	6	0.828	4.81	0.0329	-3.27	25.1	1.59
Clean Airfoil	12	1.19		0.818		14.6	
Sinusoidal A 0.025c and W 0.25c	12	0.740	38.0	0.124	52.1	5.95	59.2
Clean Airfoil	18	0.799		0.232		3.44	
Sinusoidal A 0.025c and W 0.25c	18	0.813	-1.65	0.247	6.46	3.28	4.52 (no improvement due to TLE)

The comparison of *C*_*l*_, *C*_*d*_ and L/D vs AoA for clean and the best performing sinusoidal TLE, is as shown in Table.2. The results clearly indicate that at 0, 6, 12 and 18 degree AoA, the clean airfoil constantly generates more lift and less drag than the sinusoidal TLE. The L/D ratios show that at 6° and 18° the values for both clean and sinusoidal TLE are quite close, but still the clean airfoil outperforms the sinusoidal TLE.

### 4.2 Spherical TLE

The parametric study varying the amplitude (0.025c, 0.05c and 0.075) is carried out considering spherical TLE. The *C*_*l*_ vs AoA and *C*_*d*_ vs AoA results are as shown in Figs 9 and 10. Results show that out of the three amplitudes considered, the spherical tubercle with 0.025c performed better than other two spherical TLE design.

Table 3, shows the comparison of *C*_*l*_, *C*_*d*_ and L/D vs AoA, for clean and the best performing spherical TLE. The results clearly indicate that at 0 and 12 degree AoA the clean airfoil constantly generates more lift and less drag than the spherical TLE. At 0° AoA the TLE airfoil reduced lift by 4.55% and increased drag by 17%, resulting in a decrement of L/D by 18.4%. At 6° AoA the TLE shows a lift reduction by only 3.86% and drag reduction by almost 2%, thus an overall decrease in L/D of only 1.93% is noticed. At 18° AoA the spherical TLE airfoil, outperforms the clean airfoil. The spherical TLE airfoil shows an increase in lift by 5.96% and decrease in drag by 2.67%. The overall L/D ratio at 18° shows an improvement by 6.25% due to the presence of spherical TLE. Tables 2 and 3 clearly show that both the TLE designs reduce L/D significantly. But spherical TLE performs better than sinusoidal TLE.

**Table 3:** *C*_*l*_, *C*_*d*_ and L/D for clean airfoil and spherical TLE airfoil with 0.025c and 0.25c.

Airfoil	AoA	*C*_*l*_	% decrease in lift	*C*_*d*_	% increase in drag	L/D	% decrease in L/D ratio
Clean Airfoil	0	0.353		0.0177		20.00	
Spherical A 0.025c and W 0.25c	0	0.337	4.55	0.0207	17.0	16.3	18.84
Clean Airfoil	6	0.870		0.0341		25.5	
Spherical A 0.025c and W 0.25c	6	0.836	3.86	0.0334	-1.97	25.0	1.93
Clean Airfoil	12	1.19		0.818		14.6	
Spherical A 0.025c and W 0.25c	12	0.961	19.4	0.0906	10.8	10.6	27.2
Clean Airfoil	18	0.799		0.232		3.44	
Spherical A 0.025c and W 0.25c	18	0.847	-5.96	0.232	-0.267	3.65	-6.25 (Improvement due to TLE)

## 5. Spherical tubercle working mechanism

**Fig 11:**
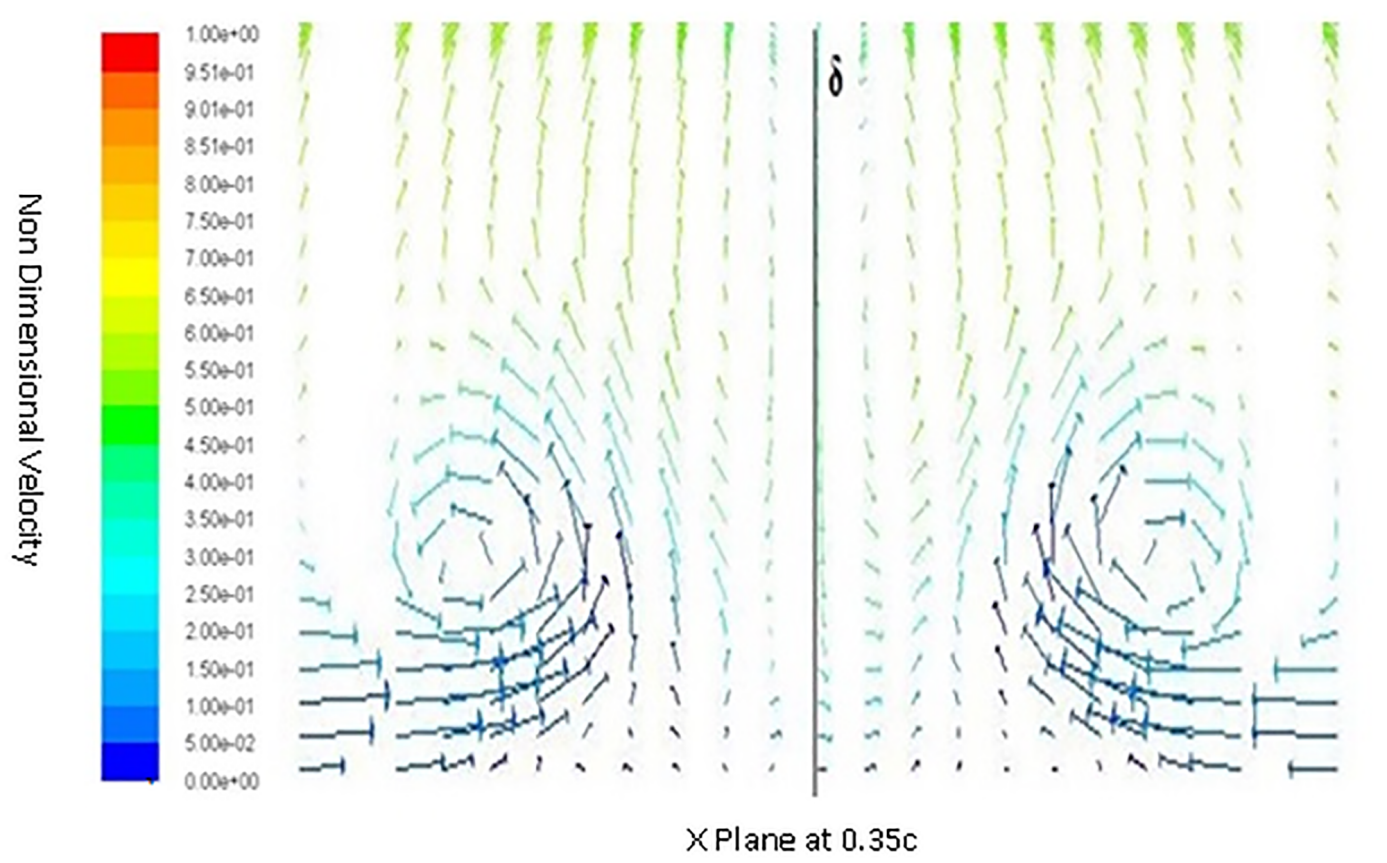
Velocity vectors X plane 0.35c downstream of airfoil.

**Fig 12:**
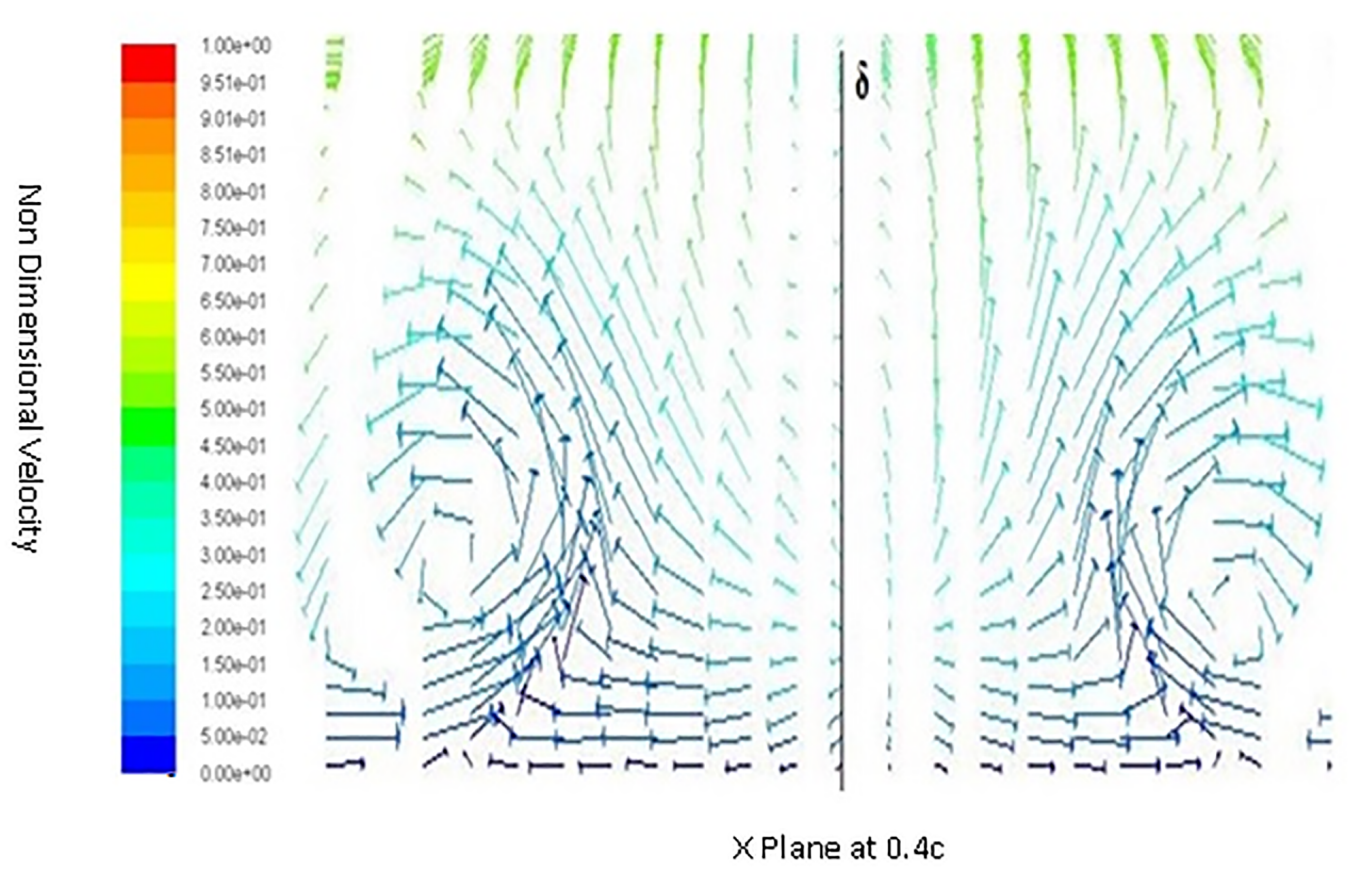
Velocity vectors X plane 0.4c downstream of airfoil.

**Fig 13:**
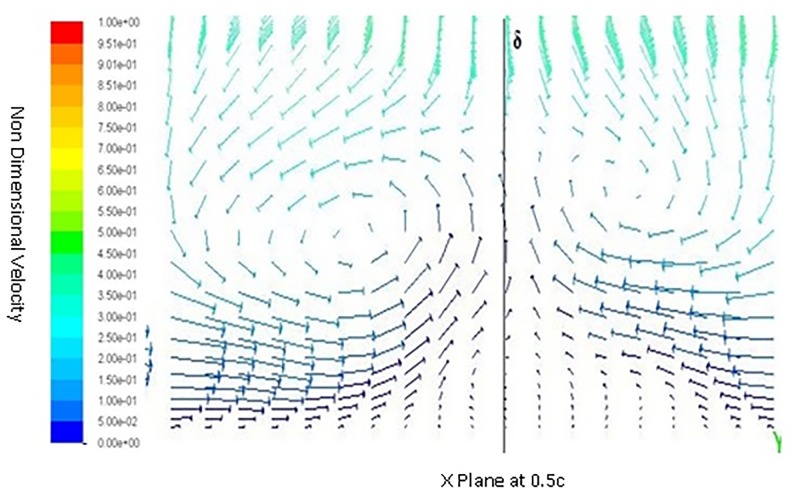
Velocity vectors X plane 0.5c downstream of airfoil.

**Fig 14:**
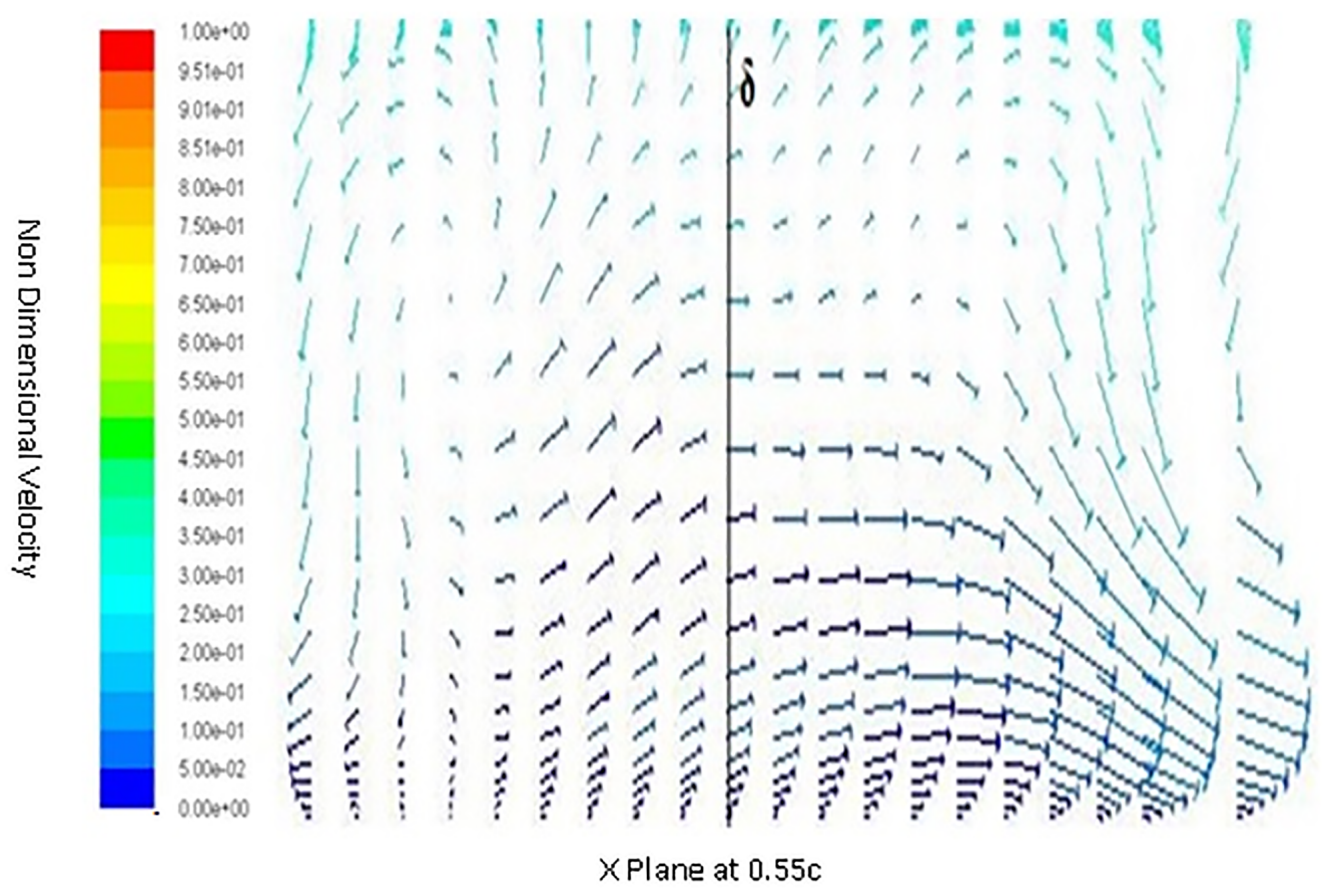
Velocity vectors X plane 0.55c downstream of airfoil.

Inorder to understand the behavior of flow around a spherical TLE, a close up of velocity vectors is plotted. The Figs 11 to 14 show velocity vectors downstream of TLE, at 0.35c, 0.4c, 0.5c and 0.55c from the leading edge of the airfoil. The velocity vectors behind the TLE, show that the TLE produces eddies which travel downstream along the chord. These eddies create flow instability further downstream and introduces vortices inside the boundary layer, thereby acting as Sub Boundary Layer Vortex Generators (SBVG). These vortices help in reattaching the BL, thereby increasing the aerodynamic performance. It is quite noticeable that no vortices were generated by TLE from the leading edge location. The TLE did induce eddies downstream, but a proper vortex formation is seen quite far from the TLE location. At 0.35c a vortex pattern is noticed, two vortices were generated within the boundary layer. A clock wise and counter clockwise vortex pattern is visible inside the BL Fig 11. The energy from the BL is sucked into the two vortices, this behavior shows that TLE act as SBVG. The size of the twin vortices grows bigger at 0.4c, as shown in Fig 12. At 0.5c these twin vortices start interacting with each other and behave similar to a standard Vortex Generator (VG). The height of the recirculation zone reaches the BL height Fig 13. At 0.55c Fig 14, the vortices lose their energy and completely disappears.

## 6. Experimental study on NACA 4415 airfoil with spherical TLE

**Fig 15:**
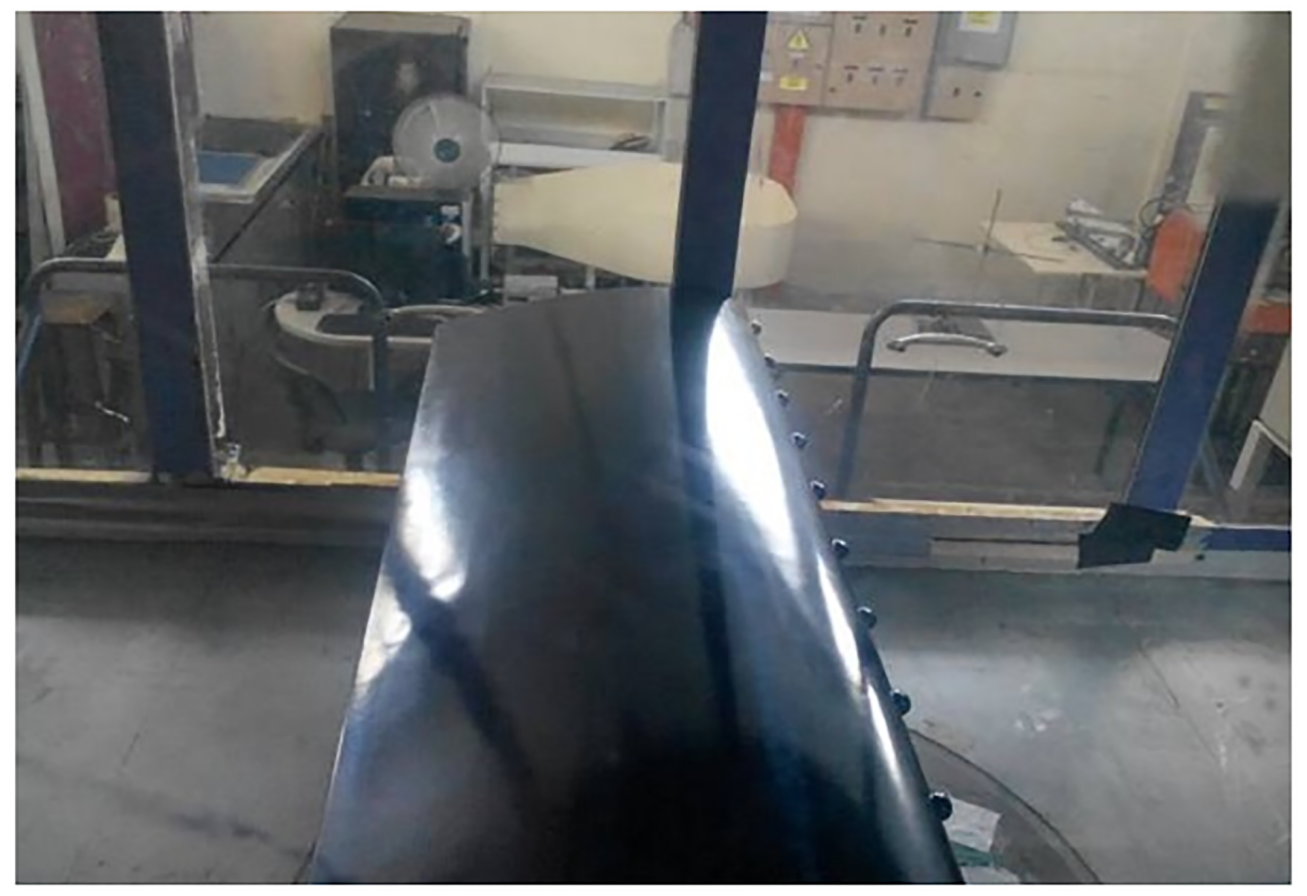
Spherical TLE airfoil in test section.

Based on the CFD parametric study results, spherical tubercle showed improved performance compared to sinusoidal TLE. Thus spherical TLE with A 0.025c and W 0.25c is fabricated as shown in Fig 15. The airfoil along with the mounting holes is designed using CATIA V5R21. The chord length of the airfoil is 0.3m and the span is 0.98m. Tubercle airfoil is fabricated using CNC machine, soft wood which is light weight and easy to fabricate is used. Fine surface finishing is obtained initially by sanding and later by painting the top and bottom surfaces. The airfoil is mounted inside the test section as shown in Fig 15.

**Fig 16:**
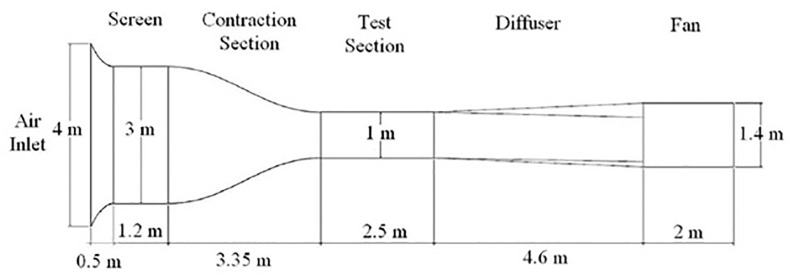
UPM open loop low speed wind tunnel [28].

The experimental study on the optimum spherical TLE configuration is carried out in the wind tunnel. The low speed wind tunnel is available at Universiti Putra Malaysia (UPM). Fig 16 shows the basic schematic of the UPM-T-1000 suction type open loop wind tunnel. The honey comb inlet houses, large screens in order to reduce the turbulence of the incoming airflow. The transparent test section is 1m × 1m × 2.5m and houses a 6 axis load balance system on which the model is mounted. The load balance is connected to the data acquisition computer. The computer is installed with Data Acquisition Reduction and Control (DARCS) software, which helps in, calibration, controlling the pitching angle and load measurements on the model.

### 6.1 Calibration

In order to ensure the correctness of the results the calibration of the force balance is carried out. Table 4 shows the range and accuracy of the force balance.

**Table 4:** Range and accuracy of the balance Wisuda [29].

Component	Range	Accuracy (%)
Lift	0 to 1000 N	± 0.1
Drag	0 to 500 N	± 0.1
Side Force	± 500 N	± 0.25
Pitching Moment	± 100Nm	± 0.25
Rolling Moment	± 50 Nm	± 0.25
Yawing Moment	± 100 Nm	± 0.25
Pitch Angle	± 400	± 0.1
Yaw Angle	± 400 to 1900	± 0.07

**Fig 17:**
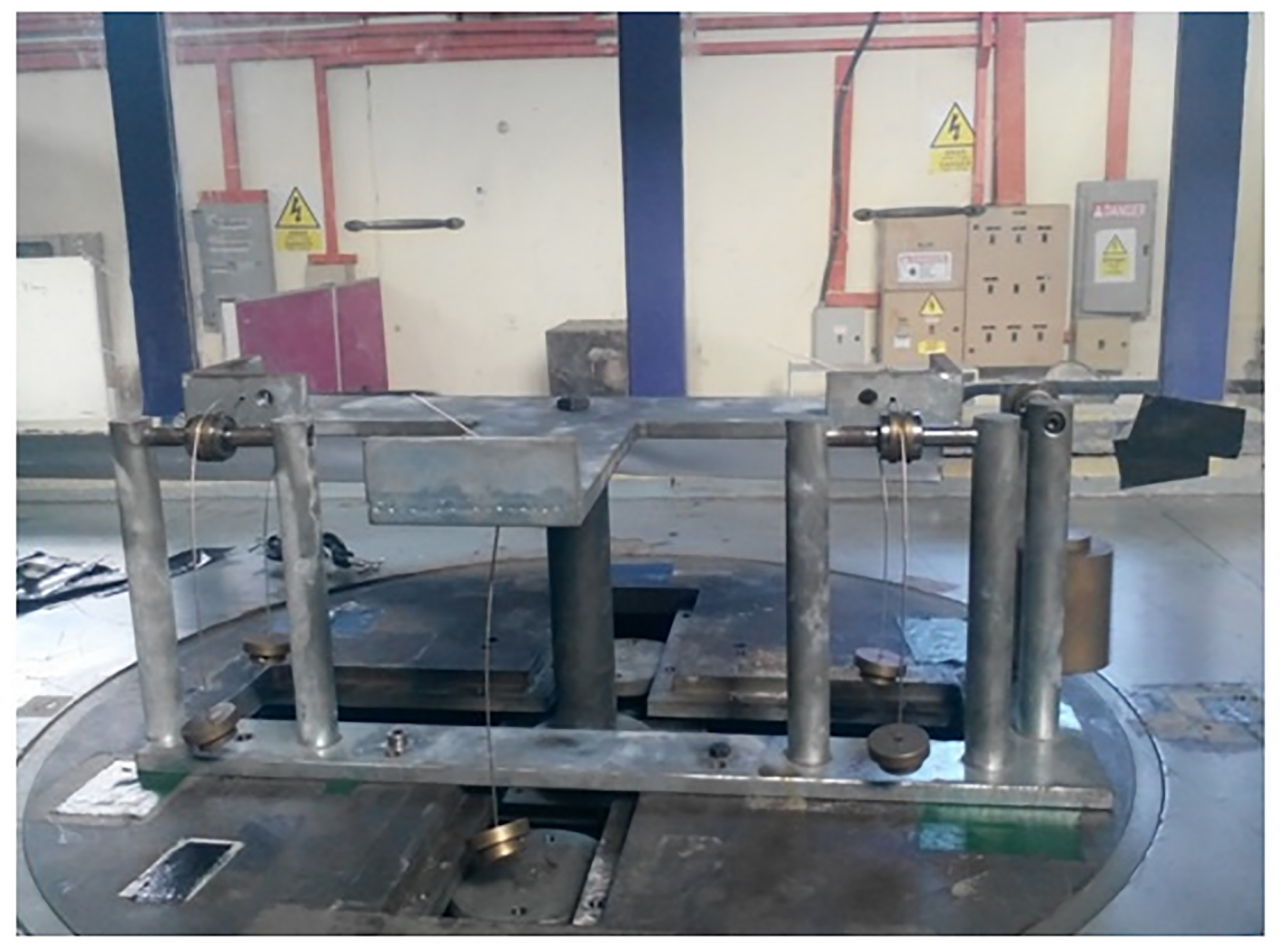
Calibration rig.

The calibration is carried by mounting the calibration rig on to the balance as shown in the Fig 17. The weight block is initially weighed on a digital scale, upon mounting the force exerted by the weight block is noted.

### 6.2 Data Acquisition System (DAQ)

The Data Acquisition System (DAQ) gathers information from the six component force balance. The balance is equipped with transducers that converts the lift, drag, moment and side forces into electrical signals. These signals are very weak of the order of millivolts which need to be amplified. An Analog to Digital convertor is used to convert the voltage to a digital signal. This digital signal is analyzed using the DARCs software.

Two pitot gauges, are used to measure the speed of air inside the tunnel. One is connected to the digital manometer. The other pitot is connected to the computer. This helps in double checking the airspeed inside the wind tunnel.

The values of *C*_*l*_, *C*_*d*_, velocity and AoA are all recorded using the DARCs software. The raw data from the DARCs software is checked for consistency. Standard procedure is followed to calculate the mean and standard deviation, for the data obtained from the wind tunnel and graphs are plotted.

### 6.3 Blockage correction

Standard blockage correction analysis, recommended in Pope and Harper [29] is applied for the aerodynamic data.

$$\begin{array}{c} C_{d0}=C_{d0u}\left(1-3\epsilon_{sb}-2\epsilon_{sb}\right) \end{array}$$

$$\begin{array}{c} \epsilon_{t}=solidblockage+wakeblockage \end{array}$$

$$\begin{array}{c} \epsilon_{t}=\epsilon_{sb}+\epsilon_{wb} \end{array}$$

The solid blockage correction for a general shape is given by

$$\begin{array}{c} \epsilon_{sb}=\frac{K_{1}V_{B}}{S^{\frac{3}{2}}} \end{array}$$

*K*_1_ = 0.74 for a horizontal modeal and 0.52 for a vertical model. S is the working section area and *V*_*B*_ is the body volume.

The wake blockage *ϵ*_*wb*_ is given by

$$\begin{array}{c} \epsilon_{wb}=\frac{c}{2h}C_{du} \end{array}$$

where c is the wing chord and *C*_*du*_ is the uncorrected drag coefficient.

### 6.4 Experimental study results and discussion

Table 5 shows the values of *C*_*l*_ and *C*_*d*_ variation from 0°AoA to 30°AoA. For each AoA three trials are carried out, and each trial consists of 15 data points, in order to determine the mean, and standard deviation.

**Table 5:** *C*_*l*_, *C*_*d*_ vs AoA.

AoA	*C*_*l*_	*C*_*d*_	L/D
0	0.471612	0.031939	14.76618
6	0.940485	0.05409	17.38756
12	1.143253	0.086542	13.21042
18	1.1474	0.224433	5.11243
24	1.027127	0.368463	2.787599
30	1.036492	0.576387	1.798256

**Fig 18:**
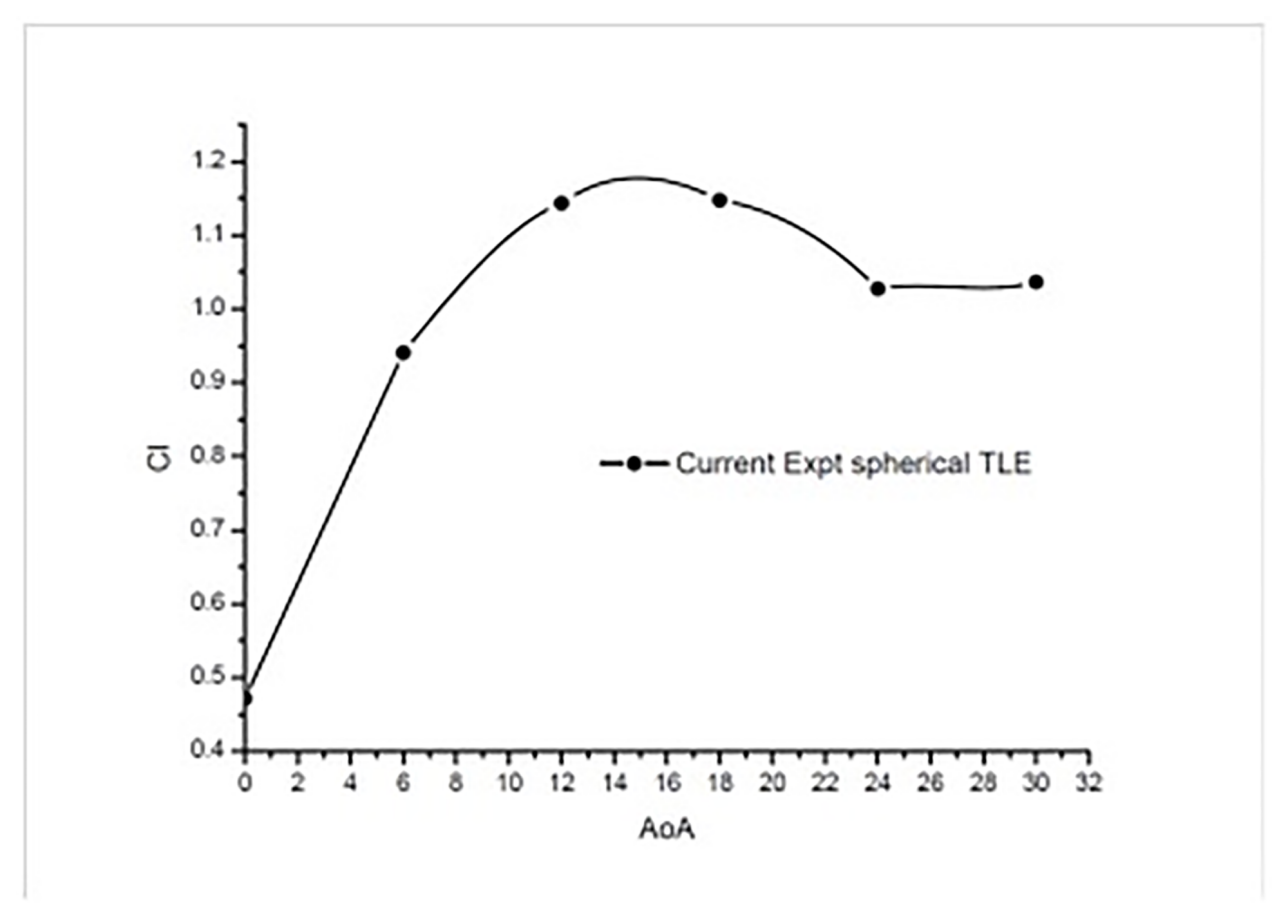
*C*_*l*_ vs AoA TLE airfoil.

The Fig 18 shows the variation of lift from 0° to 30°AoA. The TLE airfoil maintains lift over the airfoil without sudden drop at higher AoA. It can be noticed from the Table 5 that spherical TLE airfoil maintains stable lift even at higher AoA.

**Fig 19:**
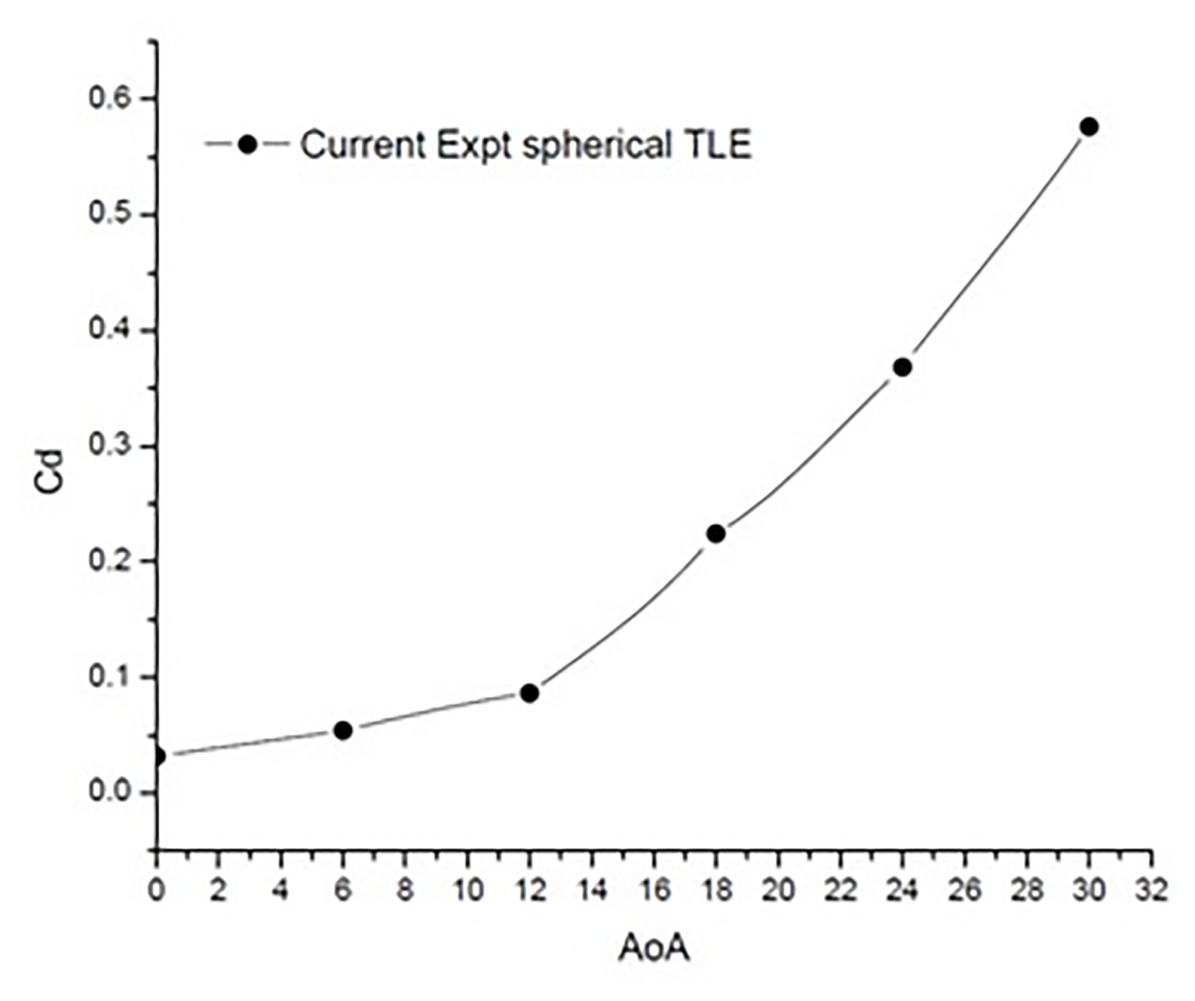
*C*_*d*_ vs AoA TLE airfoil.

Fig 19 shows the variation of *C*_*d*_ vs AoA. As expected the drag increases with increase in AoA. The values increase exponentially with increasing AoA, with a max drag of 0.576387 at 30° AoA.

**Fig 20:**
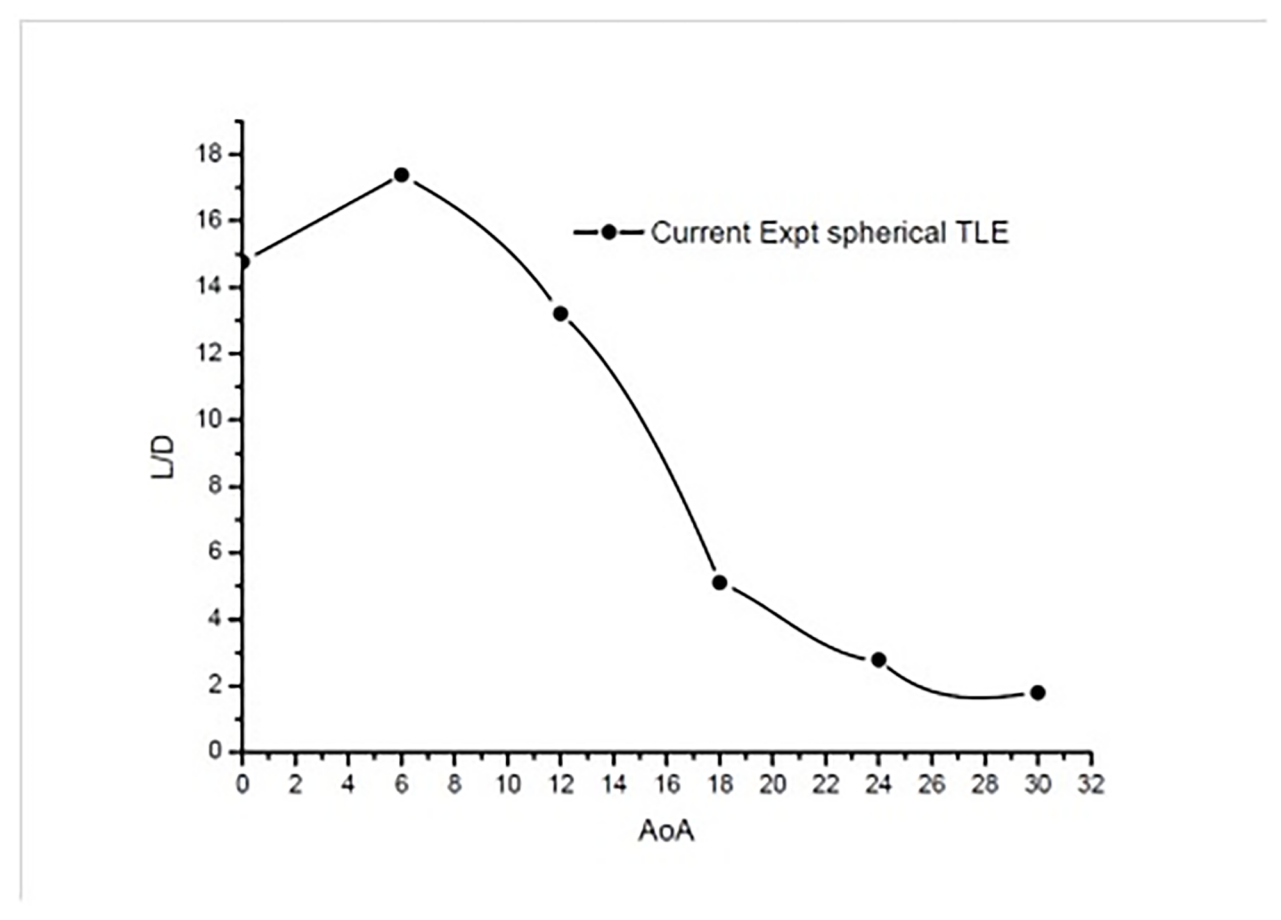
*L*/*D* vs AoA TLE airfoil.

Fig 20 shows the plot of lift to drag ratio. The TLE airfoil produces max L/D of about 17.38 at 6° AoA.

### 6.5 Comparison of TLE and clean airfoil

**Fig 21:**
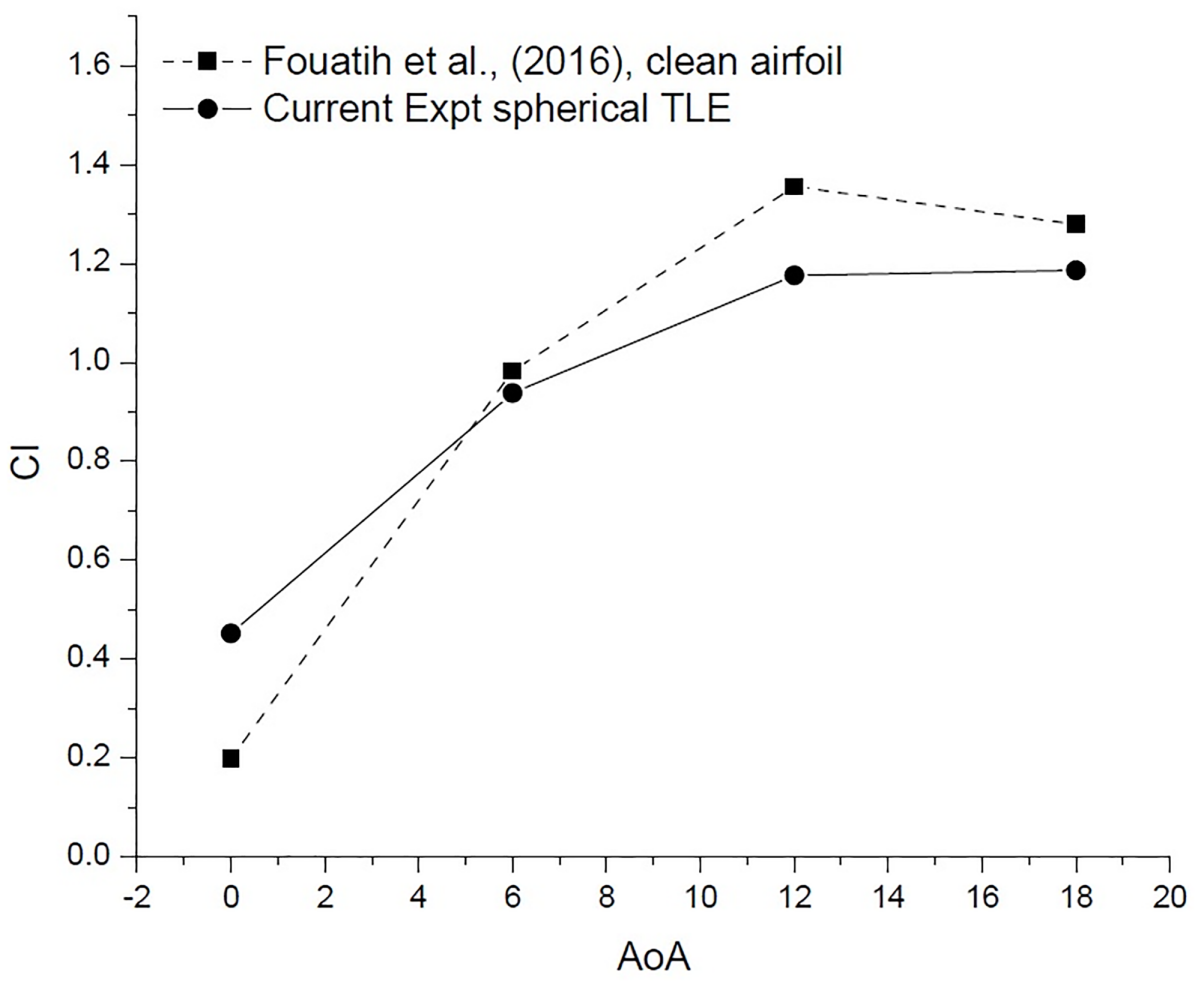
*C*_*l*_ vs AoA comparison.

The current spherical TLE experimental work is carried out at the same Reynolds numbers as in the study by Fouatih et al., [30]. The Fig 21 shows the comparison of *C*_*l*_ vs AoA for the current experimental work, with the previous work of Fouatih et al., [30] on NACA 4415. In the current experimental study, the TLE airfoil at 0° produces higher *C*_*l*_, compared to clean airfoil. Further increasing the AoA, to 6°, 12° and 18° the clean airfoil *C*_*l*_ values are better than the TLE airfoil Fig 21 [Data is provided in S1–S4 Data].

**Fig 22:**
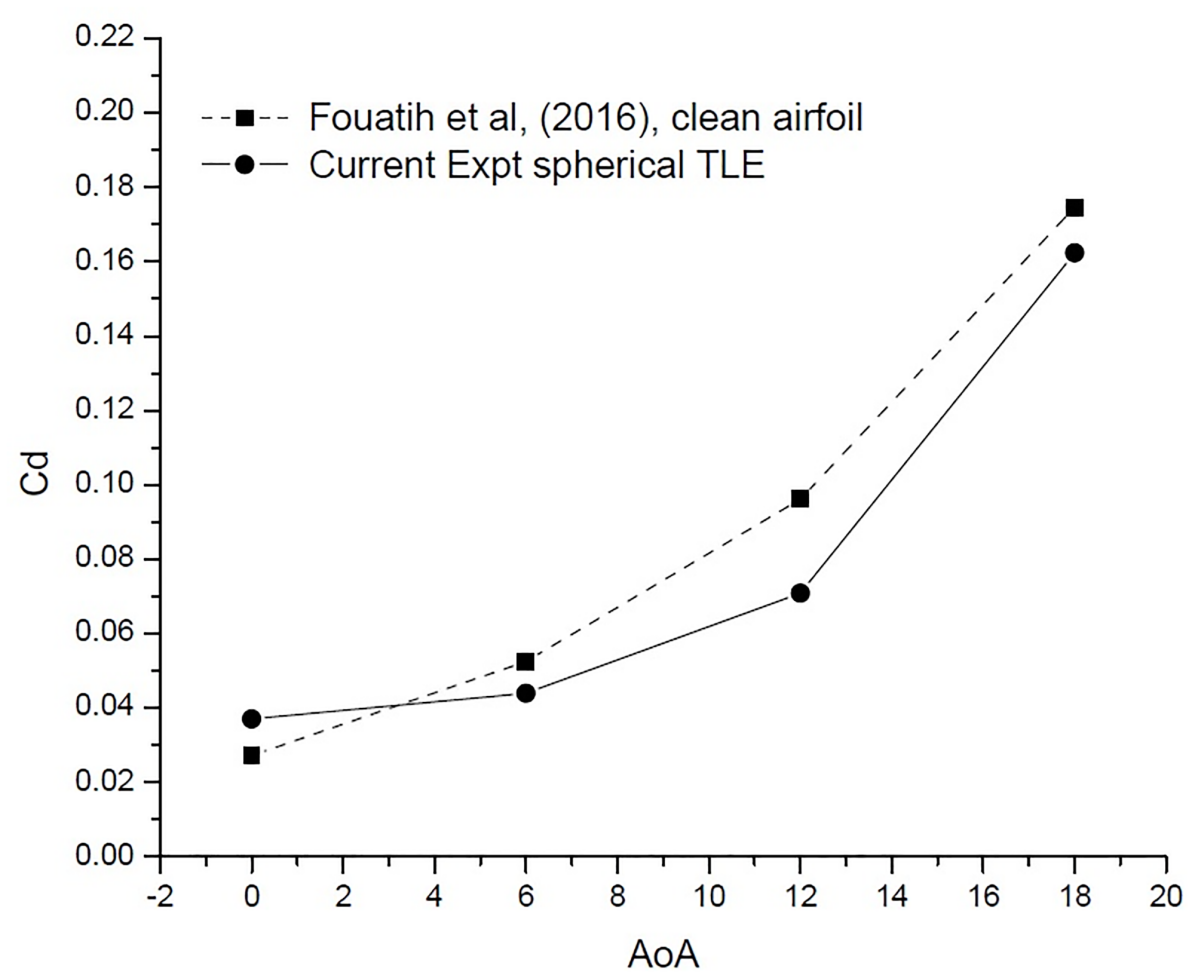
*C*_*d*_ vs AoA comparison.

Fig 22 [Data is provided in S1–S4 Data] shows the variation of *C*_*d*_ vs AoA. At 0° AoA the TLE airfoil *C*_*d*_ value is higher than the clean airfoil. The TLE airfoil outperforms the clean airfoil of Fouatih et al., [30] at other AoA. Thus these lower values of *C*_*d*_ are reflected in the overall performance improvement.

**Fig 23:**
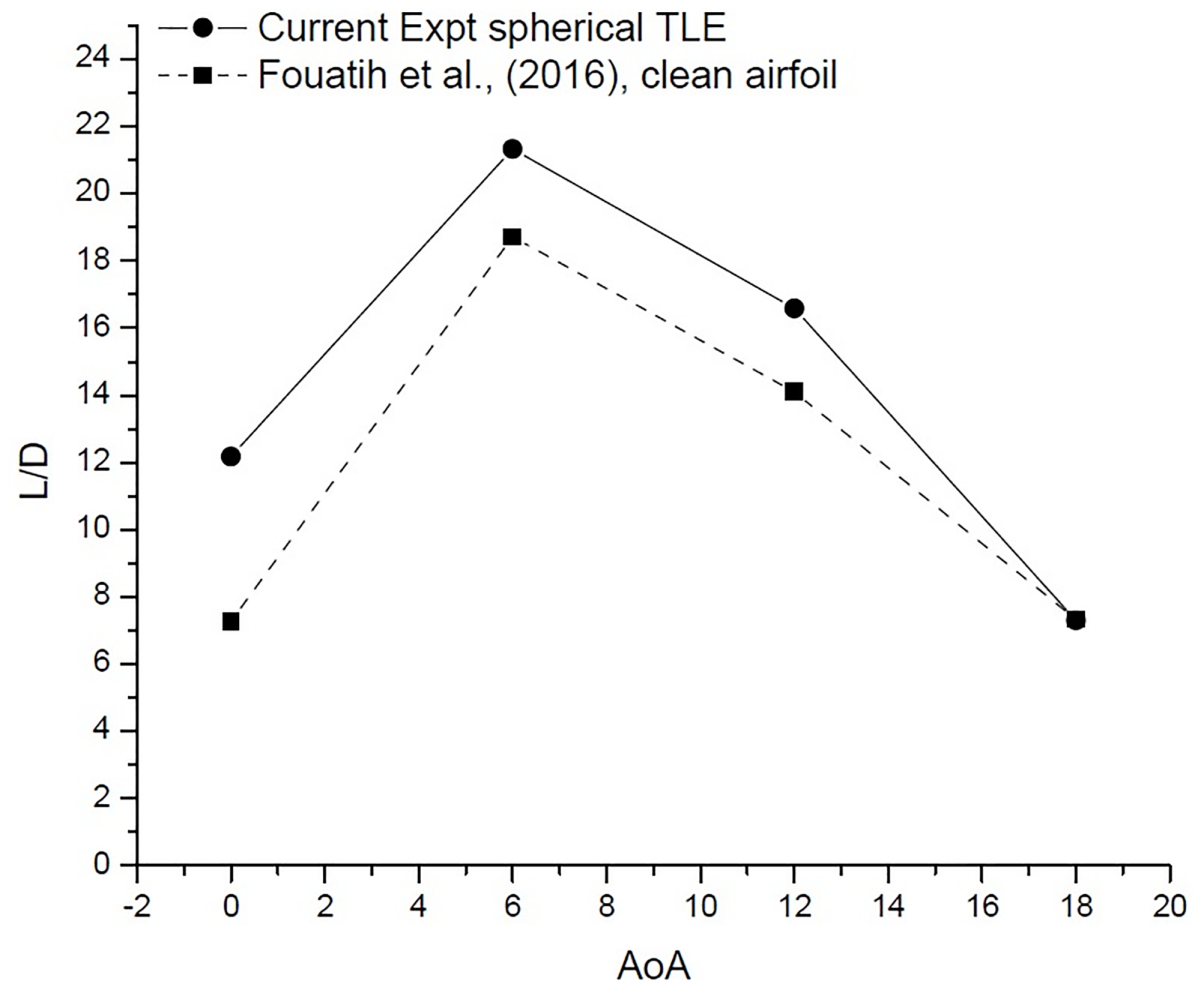
L/D vs AoA comparison.

The performance of an airfoil is measured by its L/D, TLE airfoil clearly outperforms the clean airfoil as shown in Table 6 and Fig 23 [Data is provided in S1–S4 Data]. The TLE airfoil outperforms the clean airfoil from 0° to 12°, TLE increases performance by 67.3%, 14% and 17.6%, respectively. At 12°*C*_*lmax*_ for Fouatih et al., [30] is low compared to the current TLE airfoil. At 18° the L/D ratio reduces by 3.23% compared to clean airfoil.

**Table 6:** *C*_*l*_, *C*_*d*_ and L/D for clean airfoil and spherical TLE airfoil with A 0.025c and W 0.25c.

Airfoil	AoA	*C*_*l*_	% decrease in lift	*C*_*d*_	% increase in drag	L/D ratio	% decrease in L/D ratio
Clean Airfoil	0	0.198		0.0272		7.27	
Spherical A 0.025c and W 0.25c	0	0.451	-128.09	0.037	36.3	12.17	-67.3
Clean Airfoil	6	0.982		0.052		18.71	
Spherical A 0.025c and W 0.25c	6	0.938	4.53	0.0439	-16.2	21.33	-14.0
Clean Airfoil	12	1.356		0.0961		14.10	
Spherical A 0.025c and W 0.25c	12	1.176	13.26	0.0709	-26.2	16.58	-17.6
Clean Airfoil	18	1.28		0.1745		7.33	
Spherical A 0.025c and W 0.25c	18	1.186	7.29	0.1623	-7.00	7.30	+3.23

### 6.6 Comparison of TLE and VG

In this section the result of NACA 4415 spherical TLE are compared with the results of Fouatih et al., [30] on NACA 4415 airfoil with VG. Fouatih et al., [30] studied the performance improvement by installing triangular VG. The VG’s were located at 0.3c, and the VG orientation was set at 12° AoA to the free stream. Table 7 compares the results ofthe current TLE experimental work along with the experiment of Fouatih et al., [30] on clean airfoil and airfoil with VG.

**Table 7:** *C*_*l*_, *C*_*d*_ and L/D for VG airfoil and spherical TLE airfoil with A 0.025c and W 0.25c.

Airfoil	AoA	*C*_*l*_	% decrease in lift	*C*_*d*_	% increase in drag	L/D	% decrease in L/D ratio
Spherical A 0.025c and W 0.25c	0	0.45		0.0370		12.177	
Airfoil+ VG	0	0.415	8.026	0.0233	37.050	17.8	-46.106
Spherical A 0.025c and W 0.25c	6	0.938		0.0439		21.331	
Airfoil +VG	6	1.01	-7.324	0.043	2.337	23.441	-9.893
Spherical A 0.025c and W 0.25c	12	1.176		0.070		16.587	
Airfoil + VG	12	1.48	-25.733	0.0906	-27.734	16.327	1.566
Spherical A 0.025c and W 0.25c	18	1.186		0.162		7.307	
Airfoil +VG	18	1.53	-28.886	0.203	-24.794	7.547	-3.279

**Fig 24:**
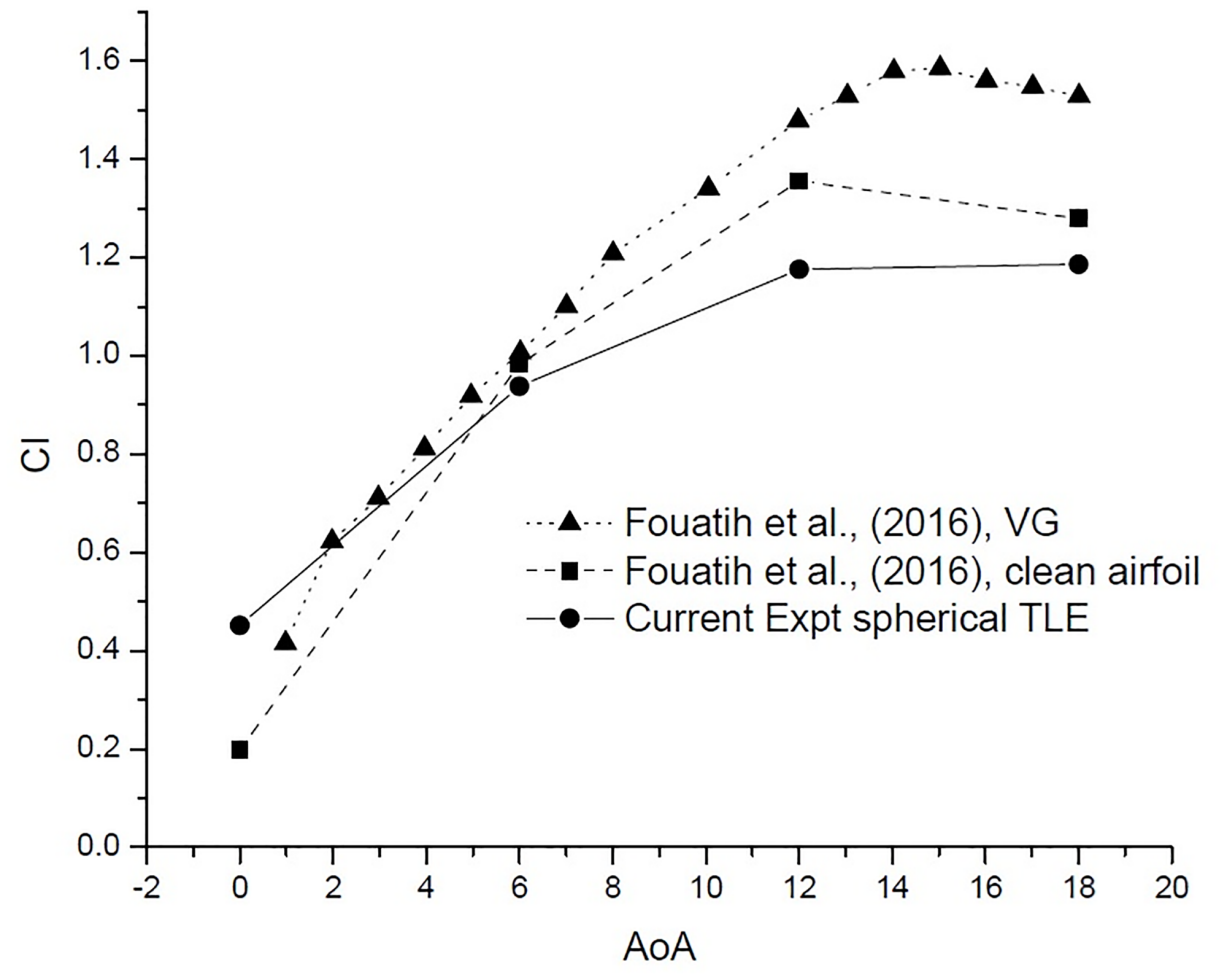
*C*_*l*_ vs AoA comparison.

**Fig 25:**
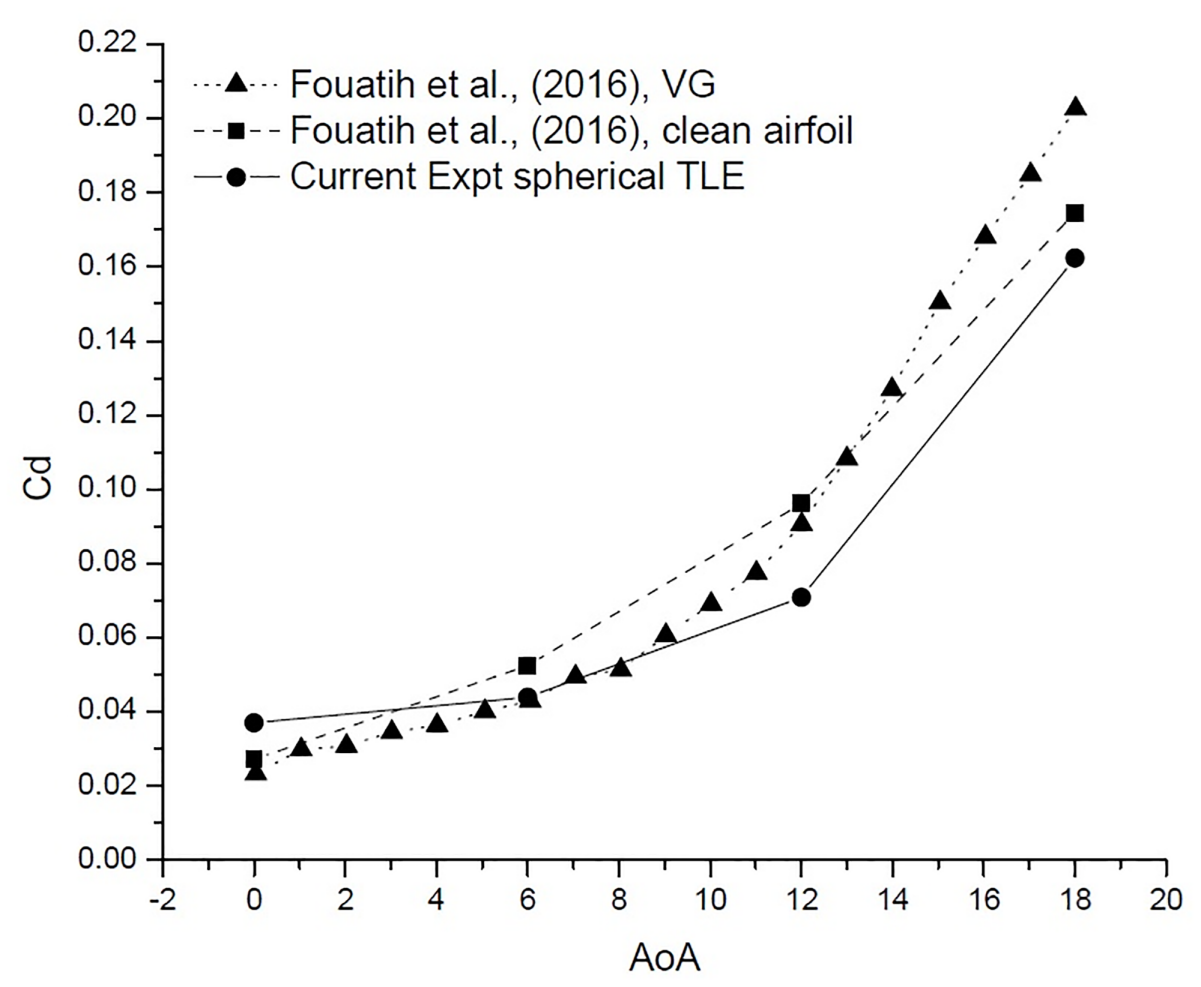
*C*_*d*_ vs AoA comparison.

Fig 24 shows the variation *C*_*l*_ vs AoA. The VG airfoil produces higher lift throughout the AoA range, except at 0°. Clean airfoil too produces higher lift in comparison with TLE airfoil. The airfoil with VG configuration produces less drag till 12°. After 12° the VG airfoil produces substantially higher drag than the clean airfoil and the TLE airfoil as shown in Fig 25. Overall TLE airfoil produces, less drag comparatively.

**Fig 26:**
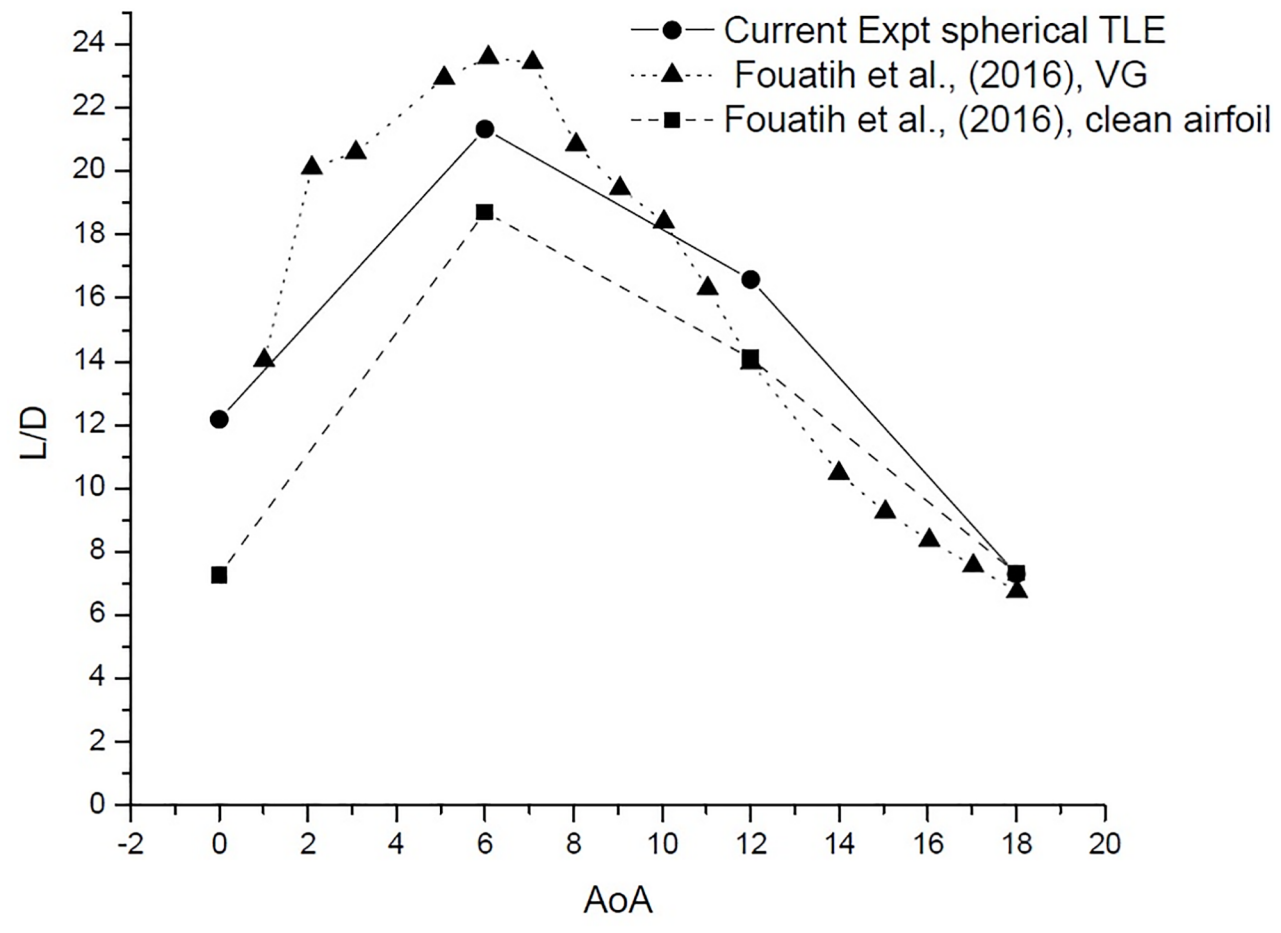
L/D vs AoA comparison.

The L/D ratio Fig 26, gives a much clearer picture. The VG airfoil performs better at low AoA below 10°. The current experimental study shows that, spherical TLE airfoil surpasses the performance of both clean airfoil and VG airfoil in between 10° to 18°.

## Conclusion

Important conclusions from the current CFD study are as follows.

1. Both in case of sinusoidal and spherical TLE, it can be observed that smaller amplitude 0.025c tubercles performed better.
2. From the above analysis, both sinusoidal and spherical TLE, reduce L/D max significantly, but the reduction of L/D max is less in case of spherical TLE than sinusoidal TLE.
3. The current study shows that, spherical TLE reduces the formation of separation bubble and outperforms the clean airfoil and sinusoidal airfoil at 18° AoA.

Important conclusions from the experimental study are as follows.

1. Results show that L/D of spherical TLE airfoil outperforms the clean airfoil.
2. Comparison of spherical TLE and VG shows that, TLE outperforms the VG airfoil and clean airfoil above 10° AoA thus improving the *C*_*lmax*_.
3. In case of CFD study the Reynolds number used was 120,000 and experimental study is carried out for Reynolds number 200,000. The results for both the cases shows that spherical tubercle behavior is Reynolds number dependent.

## Supporting information

S1 Data *C*_*l*_ and *C*_*d*_ at 0°. (XLSX)

S2 Data *C*_*l*_ and *C*_*d*_ at 6°. (XLSX)

S3 Data *C*_*l*_ and *C*_*d*_ at 12°. (XLSX)

S4 Data *C*_*l*_ and *C*_*d*_ at 18°. (XLSX)
