# Correction: CFD study on NACA 4415 airfoil implementing spherical and sinusoidal Tubercle Leading Edge

**DOI:** 10.1371/journal.pone.0188792

**Published:** 2017-11-21

**Authors:** S. M. A. Aftab, K. A. Ahmad

The grant in the Funding Section is incorrect. The correct funding information is as follows: The authors would like to acknowledge the support provided by The College of Engineering Research Center at King Saud University, for providing funds through the research grant, grant no 438/12, for the current research.
